# Role of the Mycobacterium tuberculosis ESX-4 Secretion System in Heme Iron Utilization and Pore Formation by PPE Proteins

**DOI:** 10.1128/msphere.00573-22

**Published:** 2023-02-07

**Authors:** November Sankey, Haley Merrick, Padam Singh, Janet Rogers, Amit Reddi, Steven D. Hartson, Avishek Mitra

**Affiliations:** a Department of Microbiology and Molecular Genetics, Oklahoma State University, Stillwater, Oklahoma, USA; b Department of Biochemistry and Molecular Biology, Oklahoma State University, Stillwater, Oklahoma, USA; c School of Chemistry and Biochemistry, Parker Petit Institute for Bioengineering and Biosciences, Georgia Institute of Technology, Atlanta, Georgia, USA; The University of Iowa

**Keywords:** *Mycobacterium tuberculosis*, iron acquisition, heme, type VII secretion, ESX-4, outer membrane, mycomembrane channel protein, PPE, mycomembrane, heme transport, membrane channel proteins

## Abstract

Mycobacterium tuberculosis (*Mtb*) is transmitted through aerosols and primarily colonizes within the lung. The World Health Organization estimates that *Mtb* kills ~1.4 million people every year. A key aspect that makes *Mtb* such a successful pathogen is its ability to overcome iron limitation mounted by the host immune response. In our previous studies, we have shown that *Mtb* can utilize iron from heme, the largest source of iron in the human host, and that it uses two redundant heme utilization pathways. In this study, we show that the ESX-4 type VII secretion system (T7SS) is necessary for extracellular heme uptake into the *Mtb* cell through both heme utilization pathways. ESX-4 influences the secretion of the culture filtrate proteins Rv0125 and Rv1085c, which are also necessary for efficient heme utilization. We also discovered that deletion of the alternative sigma factor SigM significantly reduced *Mtb* heme utilization through both pathways and predict that SigM is a global positive regulator of core heme utilization genes of both pathways. Finally, we present the first direct evidence that some mycobacterial PPE (proline-proline-glutamate motif) proteins of the PPE protein family are pore-forming membrane proteins. Altogether, we identified core components of both *Mtb* Heme utilization pathways that were previously unknown and identified a novel channel-forming membrane protein of *Mtb*.

**IMPORTANCE** M. tuberculosis (*Mtb*) is completely dependent on iron acquisition in the host to cause disease. The largest source of iron for *Mtb* in the human host is heme. Here, we show that the ancestral ESX-4 type VII secretion system is required for the efficient utilization of heme as a source of iron, which is an essential nutrient. This is another biological function identified for ESX-4 in *Mtb*, whose contribution to *Mtb* physiology is poorly understood. A most exciting finding is that some mycobacterial PPE (proline-proline-glutamate motif) proteins that have been implicated in the nutrient acquisition are membrane proteins that can form channels in a lipid bilayer. These observations have far-reaching implications because they support an emerging theme that PPE proteins can function as channel proteins in the outer mycomembrane for nutrient acquisition. *Mtb* has evolved a heme uptake system that is drastically different from all other known bacterial heme acquisition systems.

## INTRODUCTION

Mycobacterium tuberculosis (*Mtb*) has surpassed HIV/AIDS to become the leading cause of death worldwide and kills ~1.4 million people every year (1). *Mtb* is completely dependent on iron acquisition to successfully colonize the human host. During any infection, the host immune response can limit iron availability by complexing iron within ferritin or by removing iron with transport proteins such as transferrin (Tf) and lactoferrin (Lf), which are then stored in the form of heme (2–4). However, *Mtb* overcomes iron limitations by employing different iron acquisition mechanisms. To acquire iron from host Tf, ferritin, and Lf, *Mtb* secretes siderophores (5, 6), which are crucial for sequestering ferric iron. However, these siderophores cannot sequester iron from heme, which stores >75% of host iron (7).

In previous studies, our group (8, 9) and others (10, 11) have shown that *Mtb* utilizes heme as an iron source. Unlike other bacterial pathogens (12), *Mtb* employs novel mechanisms to capture and utilize heme. While Gram-negative pathogens use canonical β-barrel outer membrane proteins (OMPs) to capture/transport heme (12), *Mtb* instead uses cell surface PPE (proline-proline-glutamate motif) proteins (9, 13), which are found in the mycomembrane (mycobacterial outer membrane). In most pathogens, heme uptake through multiple cell surface receptors converges at an ABC transporter located in the cytoplasmic membrane (12). *Mtb* employs multiple independent pathways to transport heme across the cytoplasmic membrane. We showed that *Mtb* has at least two redundant pathways, where the genetic requirements for heme utilization (8) can be significantly altered in the presence of serum albumin. The existence of this albumin-dependent heme utilization pathway is perhaps not surprising. Since albumin strongly binds heme and is the most abundant protein in blood (14), it is likely that *Mtb* utilizes the albumin pathway for heme acquisition when blood is available. Remarkably, a very similar albumin-dependent heme acquisition mechanism has also been observed in Candida albicans (15). While these observations highlight how *Mtb* has evolved to employ unique mechanisms to acquire the essential iron micronutrient, they also demonstrate the significant gaps in our understanding of the basic underlying mechanisms of *Mtb* heme acquisition.

In this study, our primary objective was to identify unknown components of *Mtb* heme acquisition. Herein, we show that the ancestral ESX-4 type VII secretion system (T7SS) is required for efficient heme utilization. *Mtb* has five T7SS (ESX1-5). ESX-1, 3, and 5 are the most well-characterized systems playing important roles in *Mtb* virulence or iron acquisition or being essential, respectively (16). A unifying theme of ESX-1, 3, and 5 is that export of many *Mtb* PPE proteins is dependent on these systems. In contrast, ESX-2 and ESX-4 are the least studied and their functions had not been identified. Phylogenetic analysis shows that ESX-4 is the most ancestral T7SS from which all other ESX systems evolved through genetic duplication (17). In this study, we identified that the culture filtrate proteins Rv0125 and Rv1085c are required for *Mtb* heme utilization and their export is dependent on an intact ESX-4 secretion system. Furthermore, we identified that the alternative sigma factor SigM is necessary for heme utilization and is potentially a global activator of heme utilization genes. Finally, we show that some mycobacterial PPE proteins that bind heme are also channel-forming OMPs. *Mtb* PPE proteins were originally hypothesized to function in antigenic variation (18, 19), but there is an emerging hypothesis that some PPE proteins can also function as channel proteins required for nutrient uptake. Our observations provide the first direct evidence for this hypothesis.

## RESULTS

### Identification of the *Mtb* heme regulon.

**FIG 1:**
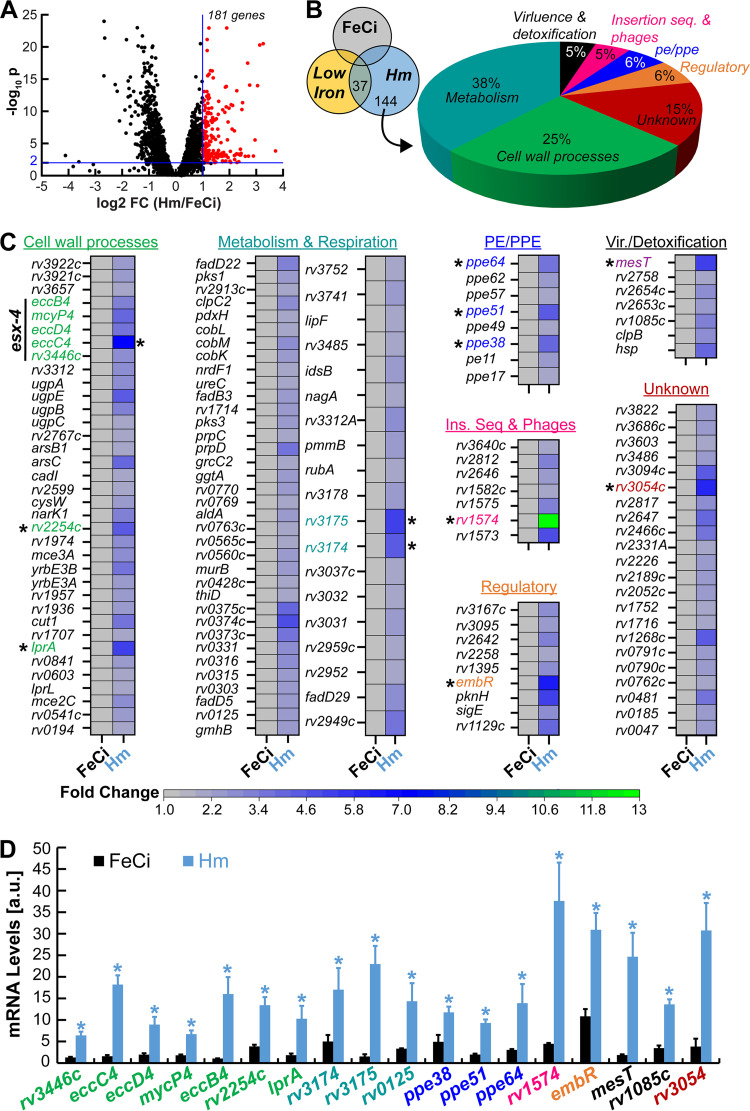
Transcriptomic analysis of Mycobacterium tuberculosis (*Mtb*) and identification of the heme regulon. (A) Volcano plot analysis between heme (Hm) and ferric citrate (FeCi) conditions where −log_10_ *q* values are plotted against log_2_ fold change (FC) for each genetic locus. Nonaxial vertical line denotes ≥2-FC of 182 genes in heme while the nonaxial horizontal line denotes the significance threshold (*q *< 0.01). (B) Category of genes that are upregulated in Mtb by >2-fold in heme compared to low iron and FeCi. (C) Heat map representing FC of the 144 heme-specific genes compared to FeCi. Light gray color of the FeCi column represents FC of 1.0 and is the same for all genes. Genes shown with asterisks were selected for validation by quantitative real-time PCR (qRT-PCR). (D) Expression levels of selected genes in FeCi or heme (Hm) determined by qRT-PCR. Error bars represent standard error of mean (SEM) values of biological triplicates; a.u., arbitrary units. Asterisks denote gene expression is significantly different in heme, compared to FeCi. Statistical significance was determined by Tukey’s honestly significant difference (HSD) following an *F* test (*P* < 0.05). Source data file is provided.

*Mtb* strain H37Rv was grown to the midexponential phase in liquid albumin-free iron-free 7H9 (7H9_-Fe_) containing either 10 μM ferric citrate (FeCi) or 10 μM heme as the sole iron source, and then we analyzed the transcriptome by RNAseq to identify genes upregulated in response to heme. Our analysis revealed that a total of 181 genes were upregulated more than 2-fold with statistical significance in heme (Fig. 1A). Interestingly, a previous study by Rodriguez et al. (20) showed that 37 (20%) of these 181 genes (Table S1 in the supplemental material) are also induced under low iron conditions. Of these 37 genes, ~57% (21) of these genes encode proteins for siderophore biosynthesis (*mbt* genes) (22), siderophore export (*mmpS4/L4/S5*) (23, 24), and siderophore import (*irtA/B*) (25–27), which are not required for heme utilization. The other 16 genes encode proteins for metabolism, putative transcriptional regulators, and proteins of unknown function. Of these low iron-induced genes, we also observed upregulation of *ppe37*, which was shown to be required for heme utilization in *Mtb* Erdman (13) but not in *Mtb* H37Rv as we demonstrated in our previous study (8). Aside from these low iron inducible genes, we identified an additional 144 genes that were upregulated in the presence of heme (Table S1; Fig. 1B and C). A significant number of these genes encode proteins for cell wall processes (25%), cell and lipid metabolism (38%), and proteins of unknown function (15%). Expression of 8 genes encoding canonical PPE and PE (proline-glutamate motif) proteins, which are known to be cell surface associated, were increased >2-fold in heme. Specifically, we observed heme-dependent upregulation of *ppe62* (Fig. 1A; Table S1), which we have shown in our previous study to be required for *Mtb* heme (Hm) utilization (9). This is in line with our previous observations and supported the experimental conditions used in this study. The other 22% of the genes encode proteins for virulence and detoxification, insertion sequence and phages, and putative transcriptional regulators. To validate our RNAseq data, we picked genes with the highest expression from each category (Fig. 1C, asterisks) and measured their mRNA levels by quantitative reverse transcriptase PCR (qRT-PCR). Expression levels of all selected genes were significantly upregulated >2-fold in heme compared to just the FeCi condition, validating our transcriptomic analysis (Fig. 1D). Altogether, our transcriptomic screen identified a heme regulon, which also includes genes that are part of the general iron stress response of *Mtb*.

10.1128/msphere.00573-22.7 TABLE S1 Transcriptomic data. Raw RNAseq expression data for all genes, genes downregulated in heme, and genes upregulated in heme. Download Table S1, XLSX file, 0.8 MB. Copyright © 2023 Sankey et al. 2023 Sankey et al. https://creativecommons.org/licenses/by/4.0/ This content is distributed under the terms of the Creative Commons Attribution 4.0 International license.

### ESX-4 is required for efficient heme iron utilization by *Mtb*.

**FIG 2:**
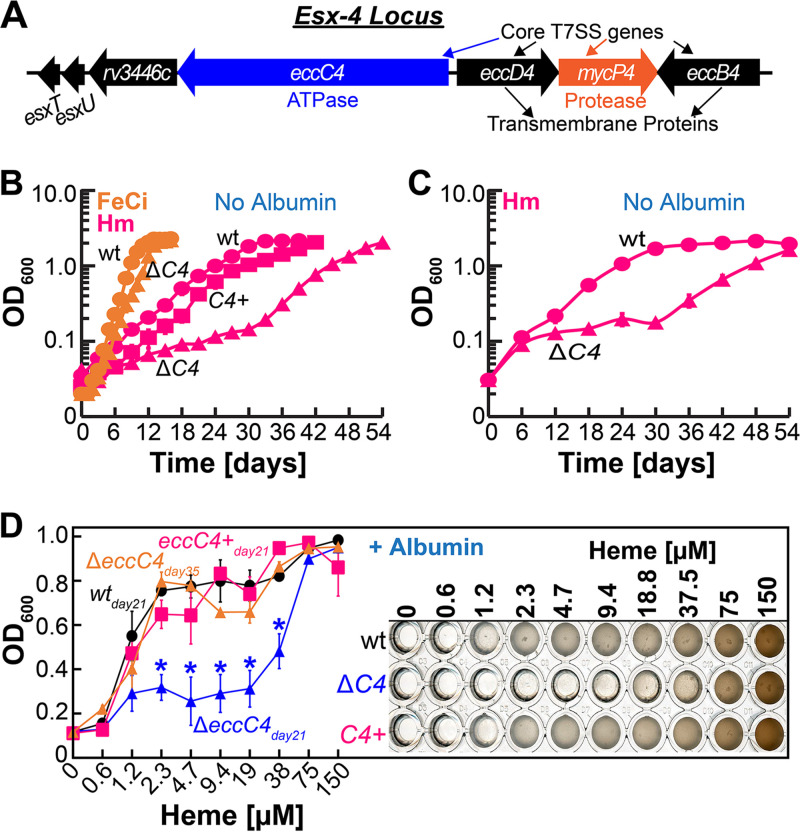
Effect of *Mtb* ESX-4 disruption on heme and albumin-heme utilization. (A) Schematic representation of the *Mtb esx-4* locus genes. (B to D) Growth of wt (circles), Δ*eccC4* (Δ*C4*, triangles), and complement (*C4+*, squares) strains. (B) Growth of strains in albumin-free iron-free 7H9 containing 10 μM FeCi (orange) or 10 μM heme (pink). (C) Growth of stationary-phase cultures of Δ*eccC4* from day 54 (Fig. 2B) inoculated into fresh albumin-free iron-free 7H9 medium containing 10 μM heme. (D) Growth of strains in liquid iron-free 7H9 containing 0.5% albumin and various concentrations of heme determined by measuring endpoint OD_600_ on day 21. Growth experiments were performed in 96-well plates (*n* = 3), where a representative image is shown on the right after 21 days of growth. Image of 96-well plate on day 35 is shown in Fig. S1D. Asterisks denote significant differences for Δ*eccC4* compared to wt or complement strains. Statistical significance was determined by Tukey’s HSD following an *F* test (*P* < 0.05). All heme medium contains 50 μM 2′2-dipyridyl (DIP) to prevent utilization of trace iron. All error bars represent SEM of biological triplicates. In many cases, error bars are smaller than marker data points. Source data file is provided.

Our transcriptomic analysis showed that the genes in the ESX-4 locus, encoding components of a T7SS (Fig. 2A), were upregulated in the presence of heme, which we verified by qRT-PCR (Fig. 1D). To determine if ESX-4 is required for *Mtb* heme utilization, we constructed an unmarked deletion mutant of *eccC4* (Fig. S1A to C), which encodes the ATPase-energizing protein for ESX-4 secretion. First, we analyzed the growth of wild-type (wt) and Δ*eccC4* in 7H9_-Fe_ containing either FeCi or heme. All heme media in our study contained 50 μM 2′2-dipyridyl (DIP) as an iron chelator to prevent trace iron utilization. Strains were always iron depleted before inoculation into the test medium. The growth of both strains is similar in FeCi reaching the stationary phase by day 12 (Fig. 2B). Heme iron utilization by *Mtb* is slower, and wt reaches the stationary phase by day 30 (Fig. 2B). Deletion of *eccC4* significantly delayed growth in heme with a long lag phase, and Δ*eccC4* starts growing exponentially around day 30. Growth impairment of Δ*eccC4* was recovered to near wt levels by complementation upon production of EccC4 from the chromosomally integrated expression vector pOAL102 (Table S3). The abrupt growth of Δ*eccC4* around day 30 (Fig. 2B) hinted a suppressor mutation could be allowing recovery. If this was the case, we hypothesized that harvesting Δ*eccC4* cells from the stationary phase and then inoculating them into fresh heme medium would allow the mutant to grow faster or at a similar rate as wt. Inoculation of stationary phase Δ*eccC4* from heme medium (day 54) into fresh heme medium shows that Δ*eccC4* still exhibits the growth delay (Fig. 2C) demonstrating that the growth recovery of Δ*eccC4* is not due to a suppressor mutation.

10.1128/msphere.00573-22.1 FIG S1 Construction of a defective ESX-4 mutant strain and characterization of mutant. (A) Schematic representation of the *Mtb esx-4* locus and PCRs performed to validate deletion of *eccC4*. Colored primer pairs show expected product length in wt *Mtb* for internal and external PCRs. (B and C) Validation PCRs of *eccC4* double crossover mutant using primers internal (B) and external (C) to *eccC4*. (D) Growth of wt, Δ*eccC4,* and complement strains in liquid iron-free 7H9 containing 0.5% albumin, 50 μM DIP, and increasing concentrations of Hm in 96-well plates on day 35. (E) Cell permeability wt (black) and the Δ*eccC4* (blue) were determined by measuring ethidium bromide accumulation. Error bars represent SEM values of biological triplicates. Source data file is provided. Growth of wt, Δ*eccC4* (Δ*C4*), and complement (*C4*+) strains on day 35 (B and C) or day 50 (D) determined by spotting dilutions on iron-free 7H10 agar plates containing 0.5% albumin (7H10A_-Fe_) with no added iron (F) or 10 μM FeCi (G) or 10 μM heme (H). Single-cell suspension of iron-depleted strains was normalized to OD_600_ 0.05 and then serially diluted and 5 μL of each dilution was spotted on agar plates. Download FIG S1, TIF file, 2.7 MB. Copyright © 2023 Sankey et al. 2023 Sankey et al. https://creativecommons.org/licenses/by/4.0/ This content is distributed under the terms of the Creative Commons Attribution 4.0 International license.

10.1128/msphere.00573-22.9 TABLE S3 Strains, plasmids, and primers used in this study. Download Table S3, DOCX file, 0.04 MB. Copyright © 2023 Sankey et al. 2023 Sankey et al. https://creativecommons.org/licenses/by/4.0/ This content is distributed under the terms of the Creative Commons Attribution 4.0 International license.

We also determined the ability of Δ*eccC4* to utilize albumin heme by growing strains in iron-free 7H9-albumin (7H9A_-Fe_) containing various concentrations of heme and measuring endpoint optical density at 600 nm (OD_600_). After 21 days, the growth of the *eccC4* mutant was significantly impaired compared to the wt and complement strains except at very high Hm concentrations (Fig. 2D). By day 35, Δ*eccC4* grew to wt levels (Fig. 2D; Fig. S1D) similar to the growth recovery observed in albumin-free heme (Fig. 2). As a solely qualitative approach, we visually examined the growth of strains by plating on self-made iron-free 7H10-albumin solid agar plates (7H10A_-Fe_). In the absence of any iron, wt and Δ*eccC4* showed residual amounts of growth (Fig. S1F). Both strains grew similarly with FeCi (Fig. S1G), but in the presence of Hm (Fig. S1H) growth of Δ*eccC4* was delayed compared to the wt and complement strains. Since cell permeability can affect the transport of hydrophobic molecules such as Hm, we determined cell permeability in Δ*eccC4* by monitoring the uptake of ethidium bromide. EtBr uptake rates were similar in both strains (Fig. S1E) suggesting that the heme growth defect is not due to altered cell permeability. Collectively, these results demonstrate that disruption of the ESX-4 system reduces the efficiency of *Mtb* heme utilization through both pathways.

### ESX-4 is required for heme uptake in *Mtb*.

ESX-4 could be involved in heme uptake by transporting heme into the cytosol or ESX-4 could be involved in heme efflux by exporting heme out of the cytosol to maintain heme homeostasis. Lack of uptake would result in the absence of iron nutrients for the cell and lack of efflux could result in intracellular heme accumulation and toxicity. Either mechanism could result in the growth defect observed in Δ*eccC4*. To differentiate these two hypotheses, we used a heme biosensor that directly responds to intracellular levels of heme.

**FIG 3:**
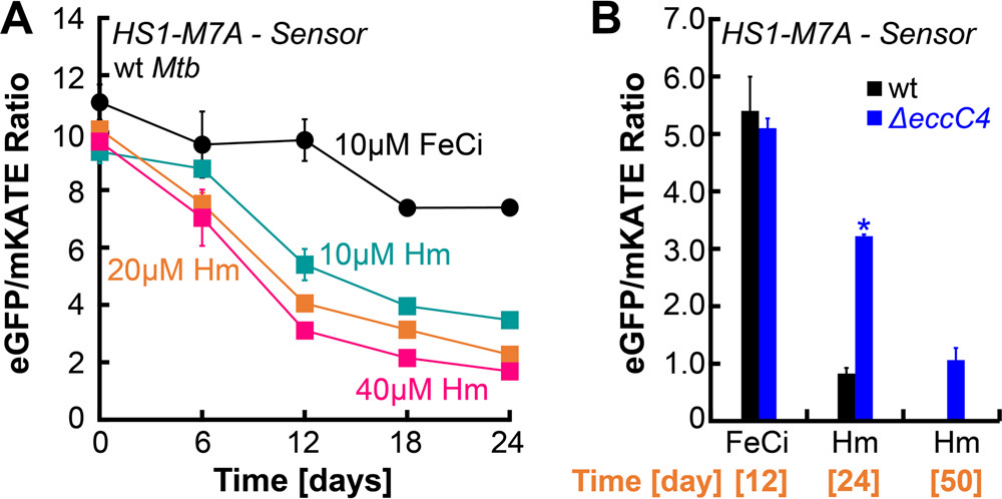
Response of intracellular heme biosensor in *Mtb*. (A) Temporal response of HS1-M7A intracellular biosensor to either 10 μM FeCi (circles) or various concentrations of heme (Hm, squares) in wt *Mtb*. (B) Temporal response of HS1-M7A biosensor to either 10 μM FeCi or 10 heme in *Mtb* (black) or Δ*eccC4* (blue). Heme medium contained 50 μM DIP to prevent utilization of trace iron. Error bars represent SEM of three biological replicates. In many cases, error bars are smaller than marker data points. Asterisks denote significant differences for Δ*eccC4* compared to wt at the specific time point. Statistical significance was determined by Tukey’s HSD following an *F* test (*P* < 0.05). Source data file is provided.

We utilized the HS1-M7A biosensor, which consists of a heme-binding domain of cytochrome b_562_ conjugated to red (mKATE) and green (eGFP) fluorescent proteins (28, 29). In the presence of heme, heme binding by the b_562_ domain quenches green fluorescence. The red fluorescence is always unaffected and serves as a useful internal standard to account for any various protein levels. The biosensor level is always reported as a green/red ratio. In the absence of heme, there is no heme binding by b_562_, green fluorescence is not quenched, and the green/red ratio is higher. Thus, we hypothesized that if ESX-4 functions in Hm uptake, then Δ*eccC4* will have lower levels of Hm relative to wt and green/red ratio in Δ*eccC4 *> wt. If ESX-4 functions in Hm efflux, then Δ*eccC4* will have higher levels of Hm relative to wt and green/red ratio in Δ*eccC4 *< wt. In a recent study, the Reddi group (30) demonstrated that the HS1-M7A biosensor is functional in *Mtb*. As such, we constructed the HS1-M7A mycobacterial episomal expression vector pOAL311, which expresses *hs1-M7A* from the constitutively active psmyc promoter (31). pOAL311 was transformed into *Mtb* and biosensor fluorescence was monitored temporally. The fluorescence ratio of the biosensor was quenched in the presence of heme in a heme dose-dependent manner demonstrating that the biosensor functioned in *Mtb* as shown before (Fig. 3A). Next, we examined biosensor fluorescence in wt and Δ*eccC4*. In FeCi, the fluorescence ratio is unchanged between wt and Δ*eccC4* (Fig. 3B). However, in heme, the fluorescence ratio in Δ*eccC4* is 3-fold higher compared to wt at day 24 suggesting significantly lower levels of heme in Δ*eccC4*, and then by day 50, the fluorescence ratio reached wt levels suggesting increased heme levels. Collectively, these results suggested that ESX-4 enabled heme uptake.

### ESX-4 does not affect PPE36 or PPE62 protein localization.

Previously, we demonstrated that PPE36 and PPE62 are two cell surface proteins required for efficient heme utilization (9). Since the export of many PPE proteins requires T7SS in *Mtb* (17), we hypothesized that disruption of ESX-4 could affect PPE36/62 export and subsequently heme utilization. We determined the subcellular localization of PPE36 and PPE62 by performing membrane fractionation experiments as we did previously (9). Fractionation experiments showed that PPE36/62 localization is unchanged in Δ*eccC4* (Fig. S2) indicating that ESX-4 does not affect PPE36/62 export.

10.1128/msphere.00573-22.2 FIG S2 Subcellular localization of PPE36 and PPE62. Subcellular localization of PPE36 and PPE62 in wt and Δ*eccC4* strains. LpqH and RNA polymerase (RNAP) were used as positive-control proteins for membrane and soluble fractions, respectively, and detected using monoclonal mouse antibodies. PPE36_HA_ and PPE62_HA_ were detected using monoclonal mouse antibodies against the HA tag. Download FIG S2, TIF file, 1.5 MB. Copyright © 2023 Sankey et al. 2023 Sankey et al. https://creativecommons.org/licenses/by/4.0/ This content is distributed under the terms of the Creative Commons Attribution 4.0 International license.

### Rv0125, Rv1085c, and SigM are required for heme utilization.

**FIG 4:**
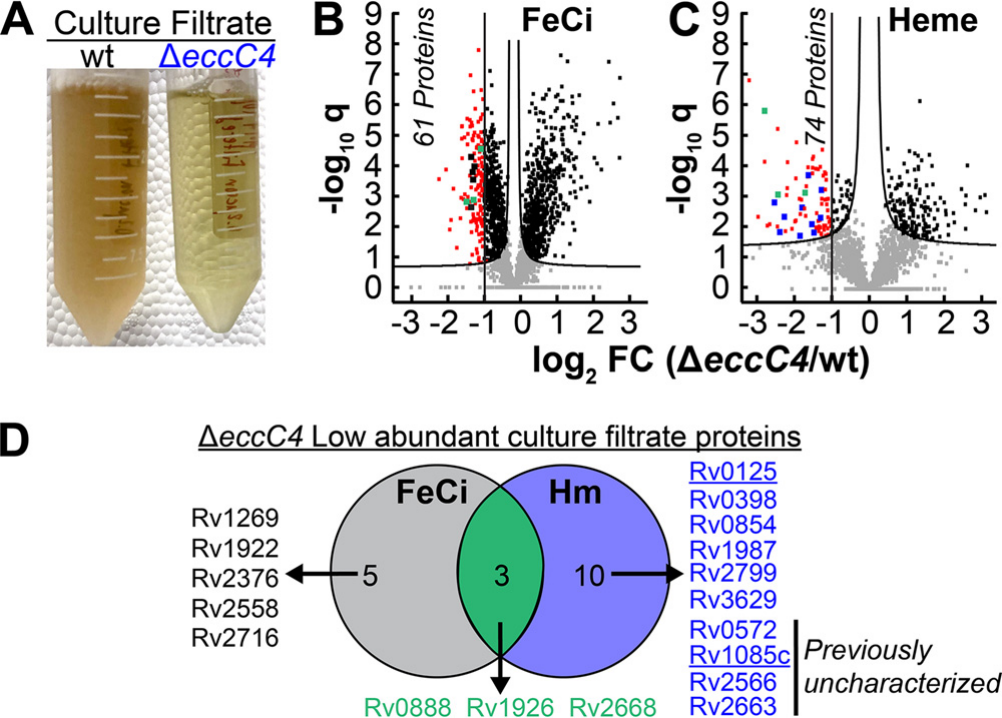
Proteomic analysis of culture filtrate protein in wild-type *Mtb* and Δ*eccC4* strains. (A) Visual analysis of culture supernatant of wt and Δ*eccC4* strains from heme medium at day 30. Picture is of heme cultures of strains on day 30 used for growth experiments in Fig. 2B. (B and C) Perseus SAM volcano plot analysis illustrating abundant proteins in wt compared to Δ*eccC4* in FeCi (B) and heme (C) conditions. The −log_10_ *q* values are plotted against log_2_ FC for each protein. Nonaxial vertical line denotes ≥2-FC of protein levels in Δ*eccC4* wt compared to wt. Asymptotic curves denotes the significance threshold (*P < *0.05). Differentially expressed proteins with statistical significance are colored. (D) Venn diagram illustrating known and unknown culture filtrate proteins identified to be in low abundance in Δ*eccC4*. Unique FeCi or heme (Hm) proteins in low abundance in Δ*eccC4* are shown colored in black dots (in panel B) and blue dots (in panel C), respectively. Rv0125 and Rv1085 underlined in blue were also identified in RNAseq analysis (Fig. 2D). Proteins in low abundance in Δ*eccC4* under both conditions are shown in green dots (in panels B and C). Source data file is provided.

While performing our heme growth analysis for wt and Δ*eccC4* in albumin-free 7H9 medium, we observed that after 30 days of growth, the culture supernatant from wt Hm medium displayed a brown-red color compared to the green color in Δ*eccC4* (Fig. 4A). Free Hm in solution has a green color and shifts to brown-red upon complexation by proteins. This suggested that Hm is complexed within some protein(s) in wt *Mtb* that were absent or in low abundance in Δ*eccC4*. Thus, we hypothesized that the ESX-4 system could be influencing the export of some culture filtrate protein(s) that is required for heme utilization. FeCi and heme supernatants were harvested for both wt and Δ*eccC4* strains as described in Methods. Culture filtrate proteins were isolated, and by mass spectrometry, we identified 2,615 proteins in both strains (Table S2). A comparison of protein levels showed that in Δ*eccC4* the levels of 1,173 proteins (Fig. 4B; Table S2) and 335 proteins (Fig. 4C; Table S2) were altered in FeCi and heme, respectively. We then focused on identifying proteins whose levels were reduced at least 2-fold or greater in Δ*eccC4*. We found that levels of 61 proteins (Fig. 4B; Table S2) and levels of 74 proteins (Fig. 4C; Table S2) were reduced >2-fold in FeCi and heme, respectively. We then used the Tuberculist (32) database to categorize proteins that were identified in *Mtb* culture filtrate in previous studies. Through this process, we identified that in Δ*eccC4* levels of 3 proteins were reduced in both FeCi and heme culture filtrate. The levels of 5 proteins were reduced only in FeCi, and 6 were reduced specifically in heme (Fig. 4D). Additionally, our proteomic analysis showed that levels of 4 proteins, which have not been previously characterized, were also reduced in the heme culture filtrate.

10.1128/msphere.00573-22.8 TABLE S2 Proteomic data. Mass spectrometer settings, raw LFQ data, all significantly low abundant proteins identified in Δ*eccC4,* and all low abundant proteins with >2-fold change identified in Δ*eccC4*Table S2, XLSX file, 0.8 MB. Copyright © 2023 Sankey et al. 2023 Sankey et al. https://creativecommons.org/licenses/by/4.0/ This content is distributed under the terms of the Creative Commons Attribution 4.0 International license.

**FIG 5:**
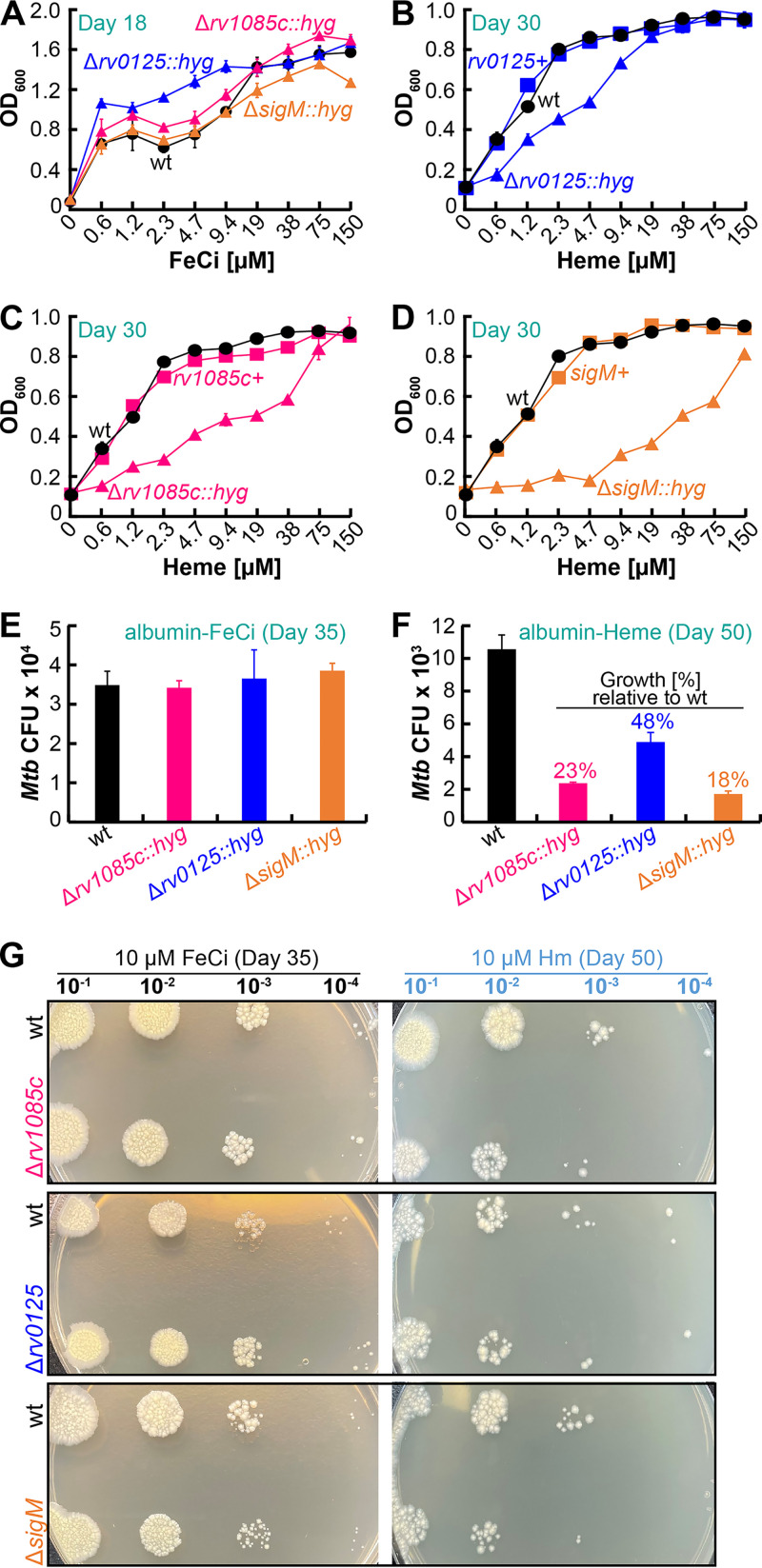
Growth of wt *Mtb* and mutant strains. (A to D) Growth of wt (black circles), mutants (colored triangles), and complement strains (colored squares). Growth was determined by measuring endpoint OD_600_ in liquid albumin-free iron-free 7H9 containing increasing concentrations of FeCi (A) at day 18 or heme (B to D) at day 30. Note: wt data points in panels B to D are the same. (E and F) Growth of strains determined by spotting dilutions on iron-free 7H10-albumin plates containing 10 μM FeCi on day 35 (E) or 10 μM heme on day 50 (F). CFU counts were determined from either 10^−3^ or 10^−4^ dilution. (F) Percent values show relative growth of mutant compared to wt. (G) Shows a representative image of agar plates used to determine CFU counts shown in panels E and F. All error bars represent SEM of biological triplicates. In many cases, error bars are smaller than marker data points. Source data file is provided.

Out of the 10 heme-specific proteins, the genes encoding Rv0125 and Rv1085c were also identified to be upregulated in wt *Mtb* (Fig. 1D) suggesting possible roles in *Mtb* heme utilization. We first constructed isogenic marked deletion mutants (Fig. S3) and then inoculated strains in medium with various iron sources and compared growth by measuring endpoint OD_600_. With FeCi, all mutants displayed similar or higher levels of growth compared to wt (Fig. 5A). In albumin-free heme, deletion of *rv0125* reduces growth to 30 to 40% (*P* < 0.05), but at heme concentrations >10 μM, there is no difference in growth compared to wt (Fig. 5B). Deletion of *rv1085c* reduces growth in heme from ~60 to 70%, and in heme concentrations >40 μM, the growth difference is abrogated (Fig. 5C). To ensure that the growth defects were specific to the gene deletion, we constructed Δ*rv0125*-and Δ*rv1085c*-complemented strains by expressing the corresponding genes from the chromosomally integrated expression vectors pOAL319 and pOAL320, respectively (Table. S3). The growth of both mutants was recovered to wt levels upon complementation with the respective gene (Fig. 5B and C).

10.1128/msphere.00573-22.3 FIG S3 Validation of *rv0125*, *rv1085c*, and *sigM* deletion mutants. Gene deletion was validated by PCR using primers external to the gene locus. Top panels show expected PCR product length (green) in wt and mutant using the same primer pair. Target gene deletion regions are highlighted in orange. Primers are described in Table S3. Bottom panels show the PCR results in wt and putative double crossover mutants. Blue tick marks indicate true marked DCOs. Download FIG S3, TIF file, 2.4 MB. Copyright © 2023 Sankey et al. 2023 Sankey et al. https://creativecommons.org/licenses/by/4.0/ This content is distributed under the terms of the Creative Commons Attribution 4.0 International license.

**FIG 6:**
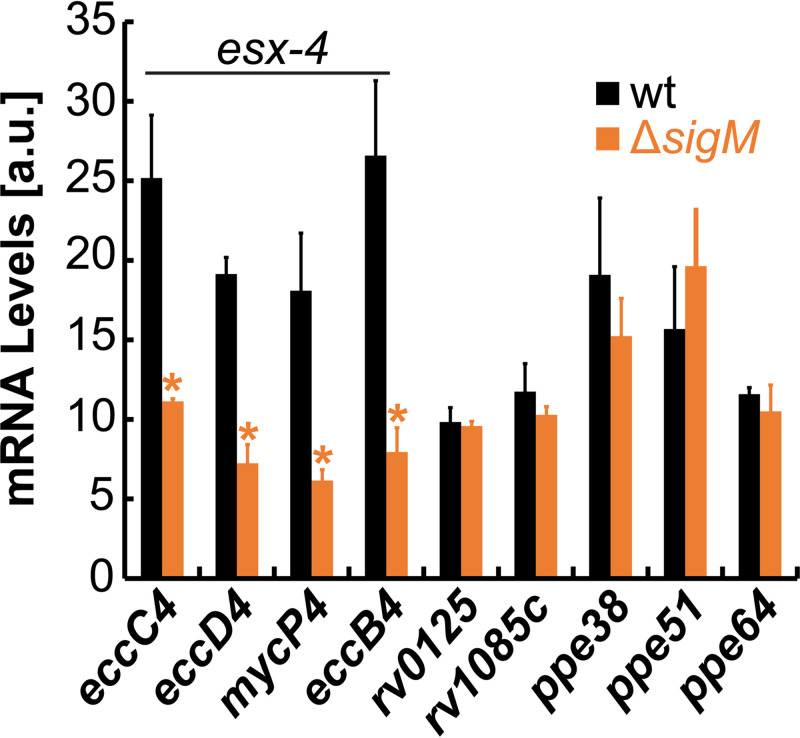
Effect of *sigM* deletion on gene expression. Expression levels of genes in wt (black) and Δ*sigM* (orange) grown in iron-free 7H9 medium containing 10 μM heme determined by qRT-PCR. Asterisks denote significant differences for Δ*sigM* compared to wt. Error bars represent SEM of biological triplicates. In many cases, error bars are smaller than marker data points. Statistical significance was determined by Tukey’s HSD following an *F* test (*P* < 0.05). Source data file is provided.

Previous studies have shown that the alternative sigma factor SigM acts as a transcriptional activator of *esx-4* locus genes (33, 34). Since ESX-4 is required for heme utilization, we hypothesized that SigM may also be required for heme utilization. We constructed a marked *sigM* deletion mutant (Fig. S3) and analyzed its growth as we did for *rv0125* and *rv1085c*. Deletion of *sigM* does not affect *Mtb* growth in FeCi (Fig. 5A) but significantly reduces growth in heme (Fig. 5D). Compared to the *rv0125* and *rv1085c* mutants, the *sigM* mutant exhibits considerably reduced growth in heme, which could be recovered to wt levels by expressing *sigM* from the chromosomally integrated expression vector pOAL321 (Table. S3). We next examined if SigM is required for activating *esx-4*, *rv0125*, or *rv1085c* by measuring gene expression. As expected, expression of all *esx-4* operon genes was significantly reduced anywhere from 2 to 4-fold in Δ*sigM* (Fig. 6). Expression of *rv0125* and *rv1085c* was unaffected in Δ*sigM* suggesting that SigM does not affect heme utilization by directly regulating transcription of *rv0125* and *rv1085c*. We also determined the ability of all mutants to utilize albumin heme by analyzing growth on solid agar plates (Fig. 5G). With FeCi, all mutants grew similarly as wt (Fig. 5E). As observed in nonalbumin heme growth (Fig. 5B to D), all mutants displayed significantly reduced growth in the presence of albumin heme (Fig. 5F). The *rv1085c* and *rv0125* mutants displayed ~23% and 48% growth compared to wt, whereas Δ*sigM* displayed ~18% growth compared to wt. Collectively, our results show that Rv0125, Rv1085c, and SigM are required for efficient heme utilization through both heme utilization pathways.

### PPE64 can bind heme and form channels in a lipid bilayer.

**FIG 7:**
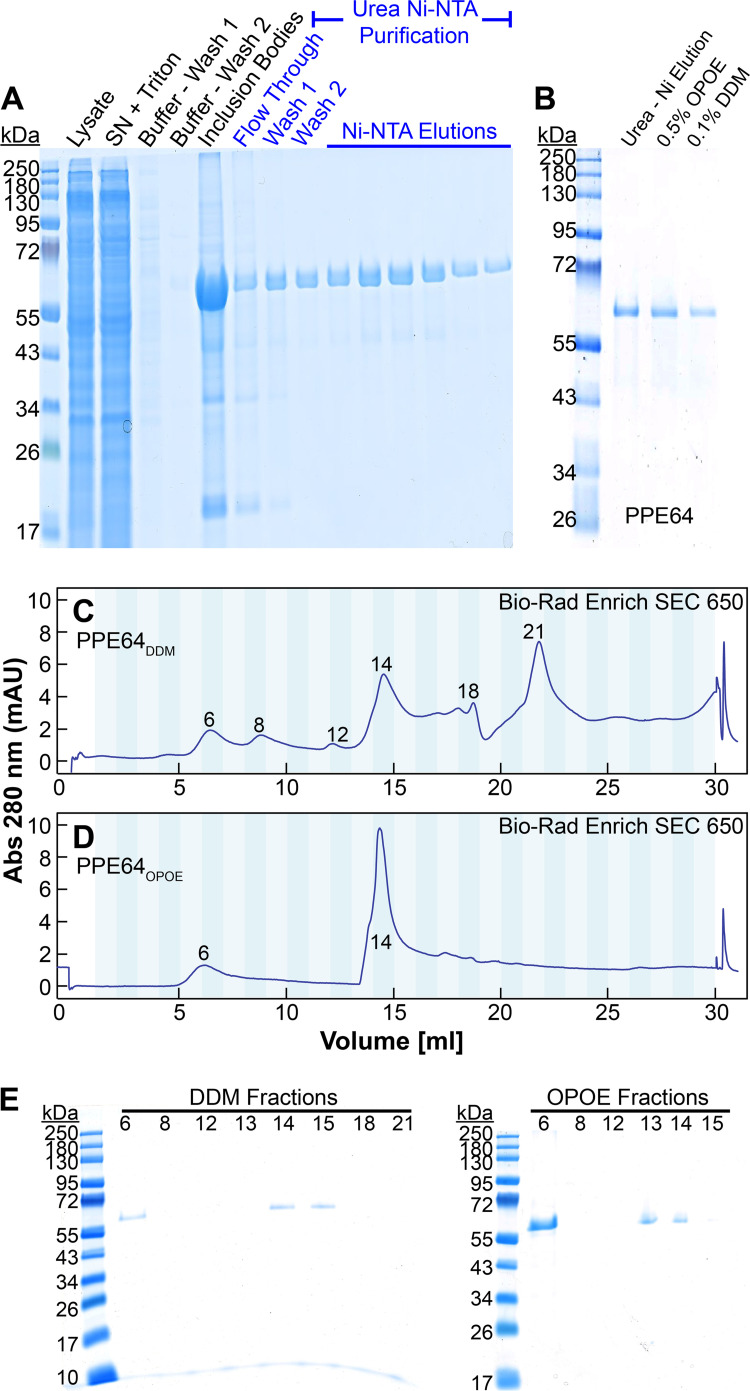
Purification of PPE64. (A) Recombinant PPE64_6His_ was purified from inclusion bodies using nickel affinity chromatography under denaturing (8 M urea) conditions. (B) Urea was dialyzed and recombinant PPE64_6His_ was then refolded in the presence of 0.5% OPOE or 0.1% DDM. (C and D) Analysis of refolded PPE64_DDM_ (C) or PPE64_OPOE_ (D) by size exclusion chromatography (SEC) using Bio-Rad Enrich SEC 650 column. Flow rate, 1 mL/min; fraction volume, 1 mL. Numbers within chromatogram show fraction number. (E) Analysis of SEC fractions by SDS-PAGE.

Our transcriptomic analysis showed that the genes encoding PPE38, PPE51, and PPE64 (Fig. 1D) were highly upregulated in heme. Since we have shown that some PPE proteins can bind heme (9), we first determined if these heme-upregulated PPE proteins can bind heme. Recombinant PPE64_6His_ (Fig. 7A), PPE51_6His_ (Fig. S4A), and PPE38_6His_ (Fig. S4E) were produced within inclusion bodies (IB) in E. coli and then purified by nickel chromatography under denaturing conditions. Removal of urea from the solution by dialysis caused proteins to rapidly precipitate, which could only be prevented in the presence of a detergent in the solution, suggesting that these PPE proteins are indeed membrane proteins. Urea was dialyzed, and PPE64_6His_ (Fig. 7B), PPE51_6His_ (Fig. S4B), and PPE38_6His_ (Fig. S4F) were refolded in the presence of 0.5% *N*-octyl-oligo-oxyethylene (OPOE) or 0.1% *n*-dodecyl-β-d-maltoside (DDM). We next examined the distribution of the refolded proteins by size exclusion chromatography (SEC) by loading equal amounts of each protein sample. With DDM, PPE64 (Fig. 7C) and PPE51 (Fig. S4C) were refolded into variable species showing the presence of aggregates and different oligomeric forms. In contrast with OPOE, PPE64 (Fig. 7D) and PPE51 (Fig. S4D) display a more uniform distribution. While PPE38 displayed a more uniform distribution in DDM (Fig. S4G) compared to OPOE (Fig. S4H), the DDM refolded proteins levels were significantly lower as observed by absorbance at 280 nm. The presence or absence of proteins in all SEC fractions for all proteins was confirmed by SDS-PAGE.

10.1128/msphere.00573-22.4 FIG S4 Purification and refolding of PPE38 and PPE51. Recombinant PPE51_6His_ (A) and PPE38_6His_ (E) were purified from inclusion bodies using nickel affinity chromatography under denaturing conditions. Urea was then dialyzed and recombinant PPE51_6His_ (B) and PPE38_6His_ (F) were then refolded in the presence of 0.5% OPOE or 0.1% DDM. Analysis of refolded PPE51_DDM_ (C), PPE51_OPOE_ (D), PPE38_DDM_ (G), and PPE38_OPOE_ (H) by size exclusion chromatography using Bio-Rad Enrich SEC 650 column. Flow rate: 1 mL/min, fraction volume: 1 mL. Numbers within chromatogram show fraction number. Download FIG S4, TIF file, 2.8 MB. Copyright © 2023 Sankey et al. 2023 Sankey et al. https://creativecommons.org/licenses/by/4.0/ This content is distributed under the terms of the Creative Commons Attribution 4.0 International license.

**FIG 8:**
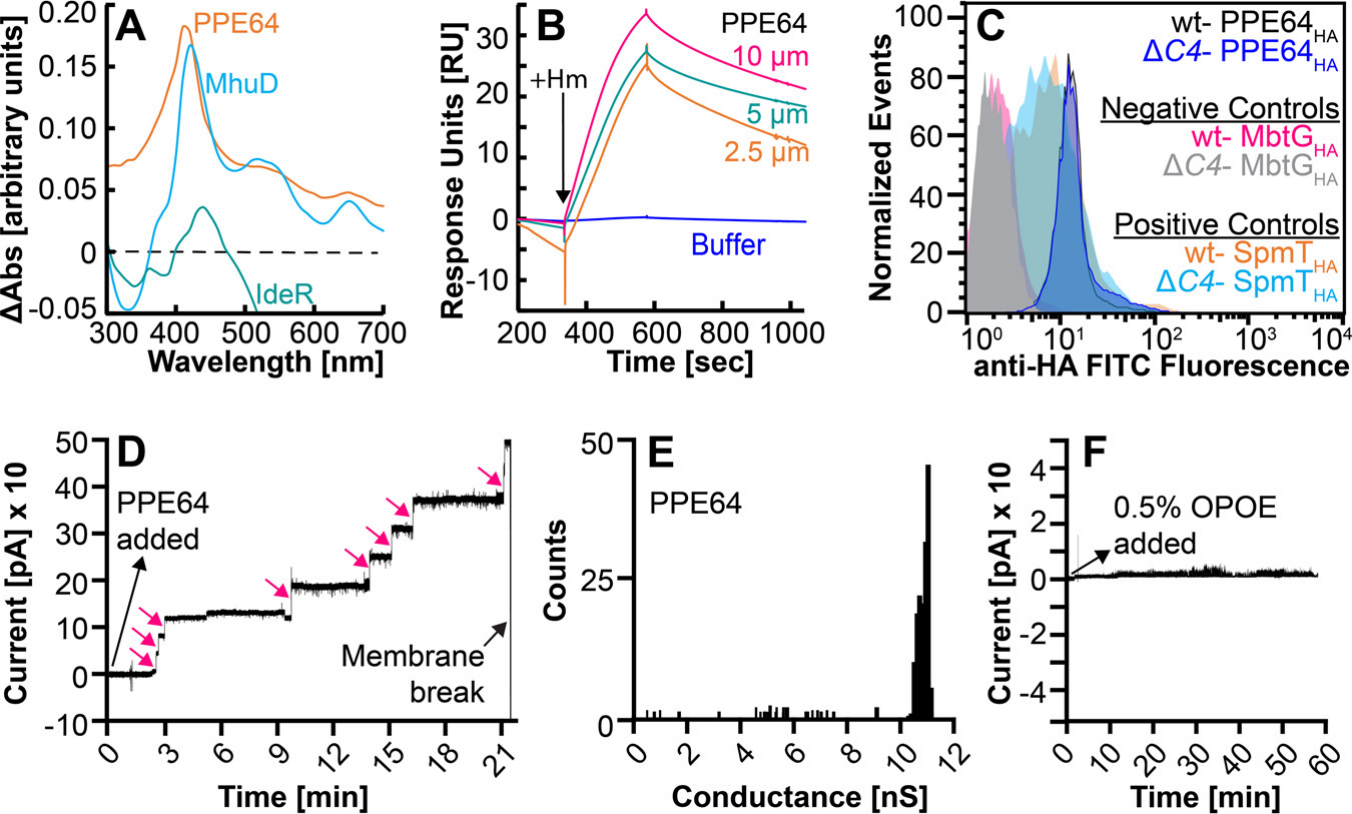
Characterization of PPE64. (A) Detection of heme binding by PPE64 through difference absorption spectroscopy. Free heme spectra were subtracted from heme-incubated protein spectra at protein concentrations of 10 μM. Spectroscopy experiments were performed a minimum of three times. (B) Heme binding by PPE64 at various heme concentrations determined by surface plasmon resonance (SPR) spectroscopy. (C) Surface accessibility of PPE64_HA_ in wt and Δ*eccC4* strains determined by flow cytometry. MbtG_HA_ (inner membrane protein) and SpmT_HA_ (outer membrane protein) were used as negative and positive controls, respectively, for surface accessibility in both strains. Source data file is provided. (D and F) Channel-forming activity of PPE64 in planar lipid bilayers. PPE64 refolded in OPOE detergent buffer was added to diphytanoyl phosphatidylcholine (DphPC) membranes with 30 mV applied potential. Current trace was recorded for PPE64 (D) and OPOE buffer (F). For panel D, each stepwise increase (pink arrows) in current trace represents a channel formation in the lipid bilayer. (E) Histogram of single-channel conductance for PPE64 collected from 5 membranes. A total of 37 pores were analyzed. The average single-channel conductance was ~11 ± 0.3 nS. Source data acquisition files (axon binary files) are too large for upload and require pClamp software for viewing. Data files will be provided upon request.

We next determined the heme binding capability of proteins by difference absorption spectroscopy. The heme spectra were subtracted from heme-protein spectra to determine the presence of the characteristic Soret peak at ~410 nm, indicative of protein-heme binding. We first used MhuD and IdeR as positive- and negative-control proteins, respectively. These proteins were purified as we did before (9), and MhuD shows the characteristic Soret peak, which is absent in IdeR (Fig. 8A). Analysis of all fractions of PPE51 (2, 9, 18, 19, 21, 35–37) (Fig. S6C and D) and PPE38 (6, 9, 14, 15, 21, 38) (Fig. S6G and H) did not show any heme binding (data not shown). However, like MhuD, only PPE64_OPOE_ fraction 14 (Fig. 7E) showed absorbance at 410 nm indicating that it binds heme (Fig. 8A). PPE64-heme binding was also validated by surface plasmon resonance (SPR) spectroscopy as we did previously (8, 9). The addition of heme to PPE64 resulted in a dose-dependent increase in signal intensity indicating heme binding (Fig. 8B). Since the export of many PPE proteins to the cell surface requires T7SS in *Mtb* (17), we determined if the export of PPE64 requires ESX-4. Thus, we determined if the cell surface accessibility of PPE64 was affected by ESX-4 disruption. *Mtb* cells expressing C-terminally HA-tagged PPE64 were harvested for the detection of protein on the whole-cell surface by flow cytometry as we did previously (9). SpmT_HA_ (surface protein) and MbtG_HA_ (inner membrane protein) were used as positive and negative controls, respectively. SpmT_HA_ was detected on the cell surface in wt and Δ*eccC4* and MbtG_HA_ detection only showed background levels of fluorescence in both strains (Fig. 8C). Cells expressing PPE64_HA_ (Fig. 8C) showed a very similar shift in signal and levels of fluorescence in both wt and Δ*eccC4*. Altogether, our data suggest that PPE64 is a cell surface heme-binding protein and its export is not dependent on an intact ESX-4 system.

10.1128/msphere.00573-22.6 FIG S6 Protein alignment. PPE62 and PPE64 belong to the PPE-MPTR subfamily of PPE proteins and share ~57% sequence similarity at the protein level. Download FIG S6, TIF file, 1.3 MB. Copyright © 2023 Sankey et al. 2023 Sankey et al. https://creativecommons.org/licenses/by/4.0/ This content is distributed under the terms of the Creative Commons Attribution 4.0 International license.

Our previous studies and others have hypothesized that cell surface PPE proteins can function as channel proteins for the transport of nutrients (8, 9, 39, 40). Since PPE64 is a heme-binding cell surface protein, we analyzed the pore-forming capability of PPE64 by monitoring protein insertion and channel formation in a lipid bilayer through electrophysiology experiments. Using diphytanoyl phosphatidylcholine (DphPC) lipids, a lipid membrane was formed in the aperture of a Delrin cup, and the baseline current trace was monitored (Fig. S5A). We first validated our lipid bilayer setup using the control protein MspA (41), which is the most abundant porin in M. smegmatis. MspA (Fig. S5F) was selectively heat extracted as described before (42) and rapidly formed channels in the bilayer (Fig. S5C). These channels showed an average channel conductance of ~4.2 ± 0.5 nS (Fig. S5D), which is in line with the reported conductance for wt MspA (43) demonstrating that our bilayer setup functions appropriately. To examine channel activity for PPE64, ~40 ng of protein was added to the *cis* compartment and the current trace was temporally monitored. All PPE64 fractions (Fig. 7C and D) from size exclusion chromatography were analyzed, but only PPE64_OPOE_ fraction 14 (Fig. 8D) led to a stepwise increase in the current trace with an average channel conductance of ~11 nS ± 0.3 (Fig. 8E), indicating that PPE64 actively inserted into the lipid bilayer and formed channels. To control for any buffer effect, OPOE buffer was added to the bilayer, which did not show any change in the current trace (Fig. 8F) or channel activity proving that the channel activity was PPE64 specific. Since PPE proteins have a highly conserved N-terminal domain (18, 19), it could be that the channel activity is a general property of all PPE proteins. To address this, we purified PPE36_6His_ (Fig. S5E) as we did before (9) and analyzed its channel-forming capability. PPE36 (Fig. S5B) did not form any channels showing that the channel activity is specific to PPE64 and also not due to any indirect effect from the 6×His tag. Our observations show that PPE64 functions in pore formation *in vitro* and argue for similar roles *in vivo*.

10.1128/msphere.00573-22.5 FIG S5 Bilayer integrity and control proteins. (A) Diphytanoyl phosphatidylcholine (DphPC) membranes were formed on a 200 μM aperture in a Delrin cuvette and the cup and chambers were filled with 1 mL of 1 M KCl electrolyte solution. Bilayer integrity and baseline noise were monitored with 30 mV applied potential for up to 15 min. (B and C) Channel-forming activity of PPE36 (B) and MspA (C) in planar lipid bilayers. (D) Histogram of single-channel conductance for MspA. The average single-channel conductance was ~4.1 ± 0.5 nS. Source data acquisition files (axon binary files) are too large for upload and require pClamp software for viewing. Data files will be provided upon request. (E) Purification of PPE36 by nickel affinity chromatography. (F) Selective heat extraction of MspA with Genapol buffer. Download FIG S5, TIF file, 3.7 MB. Copyright © 2023 Sankey et al. 2023 Sankey et al. https://creativecommons.org/licenses/by/4.0/ This content is distributed under the terms of the Creative Commons Attribution 4.0 International license.

## DISCUSSION

**FIG 9:**
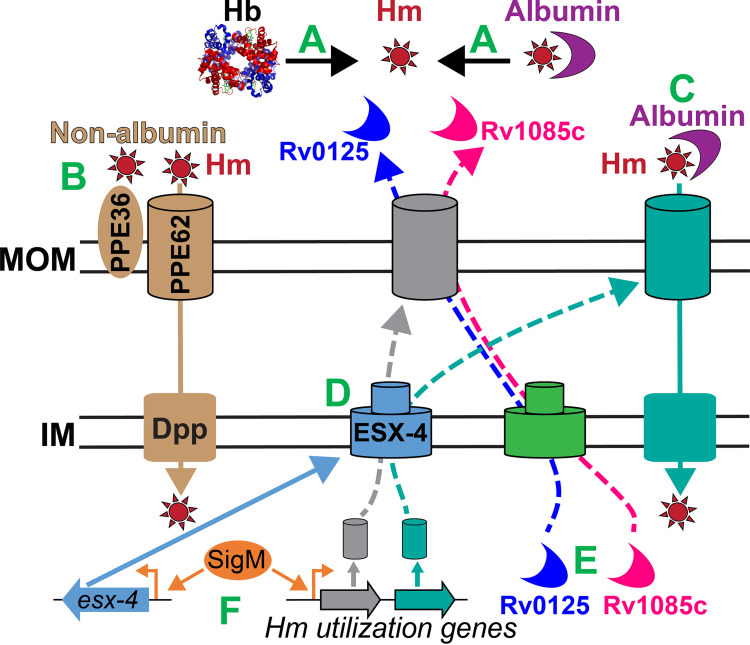
Model for *Mtb* heme utilization. (A) Heme (Hm) uptake invariably starts with removal of heme from host hemoproteins. (B) In the nonalbumin pathway, PPE36/62 functions at the cell surface, and the Dpp transporter functions at the inner membrane. (C) In another pathway, heme uptake is influenced by the presence of serum albumin. (D) The ESX-4 T7SS is involved in exporting proteins to the cell surface or extracellular milieu that are necessary for both pathways. (E) Rv0125 and Rv1085 are required for heme utilization and their export is somehow dependent on ESX-4. Rv0125/Rv1085 are likely not direct substrates of ESX-4 because they do not contain any ESX signal sequences. (F) SigM directly controls activation of *esx-4* genes and/or other unknown heme utilization genes but does not regulate expression of *rv0125* or *rv1085c*. PPE64 is an outer membrane heme binding channel protein in *Mtb* and is not shown in the model because its role in heme utilization has yet to be determined experimentally.

Previous studies have identified roles for the ESX-4 T7SS in M. smegmatis (34, 44), M. abscessus (45), and M. marinum (46, 47). More recently, in two separate studies, it was shown that ESX-4 affects the export and localization of the CpnT exotoxin in both *Mtb* and M. marinum (46, 48). Our study presents the exciting new finding that the ancestral ESX-4 T7SS is also necessary for efficient heme iron utilization through both *Mtb* heme utilization pathways (Fig. 9). The requirement of ESX-4 in heme utilization is not absolute because the *eccC4* mutant eventually reaches wt levels of growth. In M. marinum, it has been shown that there is cross-talk between ESX-4 and other T7SS such as ESX-1 and ESX-5 (47). Thus, it is possible that another T7SS compensates for ESX-4 allowing Δ*eccC4* to grow in heme. We found that *esx-3* operon genes, encoding the ESX-3 T7SS, were upregulated in the presence of heme (Table S1). It is known that ESX-3 is required for siderophore-mediated iron acquisition (49–52). Therefore, ESX-3 could be functioning as a backup system for heme utilization in the absence of ESX-4. However, this raises the question of why this compensation would take ~30 days to activate in Δ*eccC4* (Fig. 2D) for growth recovery. We also observed that numerous low-iron responsive genes (20, 53) were also upregulated in heme (Table S1). Since heme iron utilization in *Mtb* is less efficient than ferric-siderophore utilization, the induction of siderophore biosynthesis and export/import may represent a general response to slow heme iron utilization. Alternatively, the DIP iron chelator, which is membrane permeable, could be chelating some intracellular iron resulting in the activation of low iron-responsive genes in the heme medium. A reasonable argument can be made that differences in the cell permeability of wt and Δ*eccC4* could affect DIP uptake into cells exacerbating the growth phenotype seen in Δ*eccC4*. However, cell permeability of Δ*eccC4* is unchanged and as such any intracellular iron chelating effects of DIP likely remain the same in both wt and Δ*eccC4*.

By using the HS1-M7A intracellular biosensor, we show that cytosolic heme levels in wt *Mtb* reach an equilibrium around 24 days. This is reflective of the fact that the growth rate of *Mtb* is slower in heme than that in ferric iron (Fig. 2A) (8–10). Our biosensor data show that ESX-4 affects heme uptake into the cytoplasm. ESX-4 does not uptake heme directly because all T7SS systems (17) function unidirectionally to export substrates across the cytoplasmic membrane. The likeliest scenario is that ESX-4 influences the export of proteins(s) to the mycomembrane or extracellular milieu that are necessary for heme uptake. We demonstrated that ESX-4 does not affect the export of PPE36 and PPE62, two cell surface proteins that are required for heme utilization. Based on our observation of the distinct color of the wt and Δ*eccC4* heme culture filtrate, we hypothesized that some heme utilization-specific protein(s) was absent in Δ*eccC4*. In our analysis of heme culture filtrate proteins, we expected to find the proteins EsxU and EsxT as control proteins for ESX-4 secretion (Fig. 2A). EsxUT is ESAT-6/CFP-10-like proteins and genes encoding these types of proteins are found in all T7SS locus (54). The EsxUT homologs EsxAB, EsxGH, and EsxMN are secretion substrates of their cognate T7SSs Esx-1, Esx-3, and Esx-5, respectively. Since ESX-4 is involved in heme utilization and EsxUT is part of the ESX-4 locus, we expected to observe varied EsxUT levels in the wt and Δ*eccC4* heme culture filtrate. However, EsxUT was not detected under any condition in any strain in our proteomic analysis. An important point to note is that EsxAB, EsxGH, and EsxMN all have the canonical WxG and YxxxD/E type 7 secretion signal motifs. In these heterodimeric complexes, the WxG motif in one partner protein with the YxxxD/E motif in the other partner forms a bipartite type 7 recognition signal. Most importantly, the YxxxD/E motif and the 3 amino acid spacing between Y and D/E are essential for the recognition and secretion of these substrates through their cognate T7SSs (55). Even the Esx homologs in firmicutes require the WxG and *H*xxxD/Exx*h*xxx*H* motifs for secretion through their type 7B systems (56). While EsxU has the WxG motif, there is no YxxxD/E motif in EsxT. It has been suggested that EsxUT is the primary secretion substrate of ESX-4, but previous studies in *Mtb* and M. smegmatis have only examined *esxUT* gene expression through sigma factor overexpression (57, 58) and *esxUT* gene transcript levels in the context of mycobacterial distributive conjugal transfer (34, 44). While EsxUT secretion has been observed in M. abscessus (*Mab*), it should be noted that the *Mab* EsxT homolog contains the *H*xxxD/Exx*h*xxx*H* motif necessary for secretion (59). To surmise, we do not actually know if EsxUT is secreted in *Mtb*, which may explain why EsxUT was not detected in our proteomic analysis.

Our proteomic analysis showed that ESX-4 disruption decreased levels of Rv0125c and Rv1085c in the heme culture filtrate, whose corresponding genes were also identified to be upregulated in *Mtb* in the presence of heme. Rv0125 and Rv1085c are very likely not directly exported by ESX-4 because they do not contain any ESX recognition sequences (60, 61). It is more likely that ESX-4 controls the export of some other unknown protein(s) to the mycomembrane, which affects the extracellular transport of Rv0125 and Rv1085c (Fig. 9E). In an unpublished (62) proteomic analysis, the M. smegmatis Rv1085c homolog (Msmeg_5257) was also found to be in lower abundance in an *esx-4_Msmeg_* mutant supporting our proteomic analysis. We recognize that we did not directly examine the protein levels of Rv0125 and Rv1085c in wt and Δ*eccC4*, but based on the proteomic and transcriptomic precedence, we opted to characterize *rv0125* and *rv1085c* by mutational analysis and found both mutants to be partially defective in heme utilization. Rv0125 is a putative (Tuberculist) secreted trypsin-like serine protease. The active site of the trypsin serine proteases has three catalytic residues (S195, H57, and E102) (63), and Rv0125 contains similar residues S195, H57, and E113 in its predicted active site. Moreover, Rv0125 also shares sequence similarity with the secreted hemoglobin serine protease Hbp of E. coli O157:H7, which contains very similar active site residues (S207, H73, and E101) (64–67). Hbp is an autotransporter (68–70) (975 aa, ~112 kDa) consisting of a β-barrel translocator domain and a passenger domain. The passenger domain contains the protease activity that releases/binds heme by degrading hemoglobin (64, 71). Rv0125, a much smaller protein (355 aa, ~35 kDa), does not contain a translocator domain and only shares sequence similarity (33%) with the Hbp passenger domain. Thus, we hypothesize that Rv0125 may function similarly in binding and capturing heme from hemoglobin and then delivering the heme to a cell surface receptor.

Rv1085c is predicted to be a hemolysin-like protein based only on sequence similarity with hemolysins from other bacteria (Tuberculist). There is clear precedence for hemolysin function in iron utilization because bacterial pathogens use them to disrupt erythrocytic membranes to release hemoglobin to use as an iron source (72). Rv1085c is also predicted to have 7 α-helical transmembrane domains, and a reasonable argument can be made that it could be a cytoplasmic membrane protein further raising the question of why it would be present in the culture filtrate. There are two explanations for this observation. First, many pore-forming proteins of the colicin and cytolysin family also share this feature. For example, monomers of Colicin A (PDB: 1COL), which contains 10 transmembrane helices (73, 74), are first released in a soluble form that subsequently assembles into a higher-order oligomeric structure (75). Thus, it is common for pore-forming proteins such as hemolysins to contain α-helical hydrophobic helices which are commonly found in transmembrane proteins. Second, the presence of Rv1085c in culture filtrate can also be explained in the context of mycobacterial extracellular vesicle (MEV) production. It is well established that various iron/medium compositions (76) and host environments (77, 78) lead to MEV production of a different composition. For example, under iron-limiting conditions, *Mtb* releases MEVs containing the lipidic siderophore mycobactin as a mechanism to acquire ferric iron (76). MEVs are also loaded with lipids (79) that are found in the cytoplasmic membrane, which has led to the conclusion that MEVs originate from the cytoplasmic membrane. This was further corroborated by MEV proteomic analysis (80) showing that the majority of proteins are lipoproteins and proteins belonging to the cell wall, membrane function, and intermediate metabolism and respiration. A key point is that MEVs are secreted in the culture supernatant and are typically isolated using 100-kDa cutoff ultrafiltration units. In our study, we used a 3-kDa cutoff ultrafiltration unit to concentrate the whole culture filtrate, and as a result our analysis detected several of the previously identified MEV proteins (Table S2). Since heme induces *rv1085c* expression and iron availability changes MEV composition, it is conceivable that Rv1085c is released within MEVs as a mechanism of heme acquisition from host erythrocytes. This could explain why Rv1085c was found in the culture filtrate. Altogether, in our model (Fig. 9), we predict that Rv1085c may be required to lyse erythrocytes to release hemoglobin, which is then degraded by Rv0125 to release and capture heme.

We also discovered that a *sigM* mutant has a pronounced growth defect in heme (Fig. 5). This is unsurprising given that SigM is known to activate expression of the *esx-4* genes *esxT*, *esxU*, *eccC4*, and *mycP4* (57, 58). One observation is that all *esx-4* locus genes except *esxU* and *esxT* were upregulated in heme in our study. Previous studies have shown that *esxUT* expression is increased upon SigM overproduction (57, 58). Since we show that SigM and ESX-4 are required for heme utilization, we expected *esxUT* to be similarly increased as the other *esx-4* genes. There are some possible explanations for this discrepancy. First, expression analysis of *esxUT* and other *esx-4* genes in *Mtb* was primarily performed upon overproduction of SigM because native expression/level of *sigM*/SigM is not enough to induce *esx-4* genes. Second, these overexpression studies also showed that transcript levels of *esx-4* genes, which are organized in two divergent operons, are very different suggesting that there are multiple levels of regulation (transcriptional/posttranscriptional) that we do not fully understand. Third, the activation of *esx-4* genes without any overexpression to the best of our knowledge has only been observed in M. smegmatis (34, 44). Thus, the very different growth conditions of our study may explain why we did not observe increased *esxUT* expression in heme. Moreover, unchanged gene expression does not necessarily mean unchanged protein levels as EsxUT protein levels could very well be elevated by posttranslational mechanisms. Since deletion of *sigM* only reduced the expression of *esx4* genes (Fig. 6) but did not eliminate their expression, this suggests that SigM may act as an activator of other unknown heme utilization genes beyond the *esx-4* locus (Fig. 9F). Since SigM is required for both heme utilization pathways, this gives us the first potential handle to identify components of the albumin-heme pathway. A future direction of exploration will be to examine the transcriptomic profile of Δ*sigM* to identify genes specifically expressed in albumin heme. While these findings help us improve our understanding of *Mtb* heme utilization (Fig. 9), clearly further experimentation is needed to determine the exact mechanisms of how ESX-4, Rv0125, Rv1085c, and SigM function in *Mtb* heme utilization.

Our transcriptomic analysis showed that the genes encoding PPE38, PPE51, and PPE64 were significantly upregulated in heme. This was significant because in our previous studies we showed that some *Mtb* PPE proteins are required for heme utilization (9). We determined that recombinant PPE38 and PPE51, which were extracted and refolded from inclusion bodies, do not exhibit any heme binding. This suggests that these proteins either do not bind heme or maybe they are in a folded state that does not support heme binding. Clearly, further experimental work with native proteins and through mutational analysis is necessary to determine if these proteins have any role in heme iron utilization. However, we identified that PPE64 is a cell surface protein (Fig. 8C) and demonstrated using two spectroscopy approaches that it binds Hm (Fig. 8A and B). These observations are perhaps not entirely surprising because PPE64 shares 57% sequence (Fig. S6) similarity with PPE62, which we showed is also a cell surface protein that binds heme and is required for heme utilization (9). Since deletion of *ppe62* only partially affects *Mtb* heme utilization, it is possible that PPE64 could be a redundant functioning partner of PPE62. We fully recognize that a drawback in our study is that we have yet to determine if PPE64 contributes to *Mtb* heme utilization through mutational analysis. However, an exciting finding of our study is that PPE64 is a channel-forming membrane protein as demonstrated through lipid bilayer analysis (Fig. 8E and F). To the best of our knowledge, this is the first direct evidence of channel activity by a PPE protein. Using PPE36 and MspA as negative and positive controls, respectively, we showed that PPE64 actively forms channels in a lipid bilayer (Fig. 8E and F). The channel conductance of PPE64 is ~11 nS, which is vastly different from other mycobacterial channel proteins such as MspA (~5 nS) (43), MctB (4.5 nS) (81), and CpnT (1.36 nS) (82) or nonmycobacterial porins such as OmpA (50 to 320 pS) (83), OmpC/F (0.47/0.84 nS) (84), OmpG (0.8 nS) (85), or HiB (0.85 nS) (86). An important point to note is that we do not know if the channel conductance of refolded PPE64 is fully representative of native PPE64. However, the fact that we specifically observe channel activity only from fraction 14 of OPOE refolded PPE64 suggests that the protein is in a folded state that supports channel formation in a lipid bilayer. Altogether, our observations support an emerging theme that mycobacterial PPE proteins can function as channel proteins in the mycomembrane for the transport of different molecules. We hypothesize that the drastically different mycomembrane architecture (87) has directed *Mtb* evolution to use PPE proteins as channel proteins. Some PPE proteins have a cognate PE (proline-glutamate motif) protein partner (18, 19), and these *ppe-pe* genes are often in tandem in the chromosome. However, not all PPE proteins have or even need an interacting PE partner. Our lipid bilayer experiments show that PPE64 is in a folded state that supports channel formation without requiring a PE partner. To date, crystal structures of only smaller PPE proteins with a PE protein partner have been resolved (60, 88), leaving a huge gap in our knowledge of how channel-forming PPE proteins function. Since mycobacterial PPE proteins are primarily found in pathogenic mycobacteria, understanding their mechanisms in nutrient acquisition, particularly in iron acquisition, makes them prime candidates for developing highly targeted chemotherapy. In conclusion, we believe our study presents exciting new findings that will open new avenues in mycobacterial research.

## MATERIALS AND METHODS

### Bacterial strains, growth media, and molecules.

Wild-type Mycobacterium tuberculosis H37Rv and its derivative strains were grown in Middlebrook liquid 7H9 or solid 7H10 medium supplemented with 10% ADS (8.5 g/L NaCl, 20 g/L dextrose, and 50 g/L bovine albumin fraction V), 0.5% glycerol, 0.2% Casamino Acids (CAA), and 0.02% tyloxapol. This fully supplemented medium is referred to as complete 7H9 medium from hereon. Escherichia coli DH5α was grown in either LB medium containing appropriate antibiotics at 37°C with shaking at 200 rpm. The following antibiotics were used when required: ampicillin (Amp) at 100 μg/mL for E. coli, kanamycin (Kan) at 30 μg/mL for mycobacteria and 50 μg/mL for E. coli, hygromycin (Hyg) at 200 μg/mL for E. coli, and 50 μg/mL for mycobacteria.

### Preparation of iron-free medium.

7H9 is a defined medium whose composition is known and by default contains 150 μM ferric citrate. All components except ferric citrate were dissolved in millipore water in acid-washed beakers to prepare the base iron-free 7H9 medium (7H9_-Fe_). Lyophilized albumin was added to this base medium to prepare the base iron-free albumin-7H9 medium (7H9A_-Fe_). The medium was then filter sterilized through a 0.2-μm filter. Freshly made ferric citrate or hemin solution was added to either base medium to prepare the specific iron-containing medium. Hemin solutions were prepared in Tris buffer as described previously (35). 2,2-Dipyridyl (DIP) iron chelator solutions were prepared in DMSO and added to freshly made hemin medium.

### Growth experiments for determining iron utilization.

Unless specified, all liquid cultures were grown in sealed square PETG bottles with shaking at 120 rpm, all incubation was done at 37°C, and all liquid and solid growth medium experiments were performed in triplicate. Strains were first grown to the midexponential phase in complete 7H9, then washed in sterile PBS containing tyloxapol, and then iron depleted for 10 generations in iron-free 7H9 medium containing ADS, glycerol, CAA, and tyloxapol. This iron-depletion protocol was strictly performed before all growth experiments. Iron-depleted cells were passed through a 5.0-μm filter to obtain a single cell suspension, which was then used to inoculate iron-free liquid 7H9 or plate on iron-free solid 7H10 agar containing specific iron sources as mentioned in the main text. For plating on solid agar plates (Fig. 5E and F; Fig. S1F to H), single cell suspension was prepared at OD_600_ 0.05, which was serial diluted, and 5 μL of each dilution was spotted on agar plates. Solid agar medium was grown for 35 to 50 days as described in the figure legends. Bovine serum albumin was added to the medium to a final concentration of 0.5% wt/vol (75 μM) for albumin growth experiments. Unless specified, all heme media (liquid and solid) in our study contained 50 μM the iron chelator 2,2-dipyridyl (DIP). For growth experiments in Fig. 2B and C, strains were inoculated in 30 mL of medium at an initial optical density of OD_600_ 0.01. For growth experiments in Fig. 2D, strains were inoculated in a final volume of 200 μL in 96-well plates at an initial optical density of OD_600_ 0.001 in wells containing various concentrations of heme. The growth of strains was determined by measuring endpoint OD_600_ on days 21 and 35. For growth experiments in Fig. 5A to D, strains were inoculated in a final volume of 200 μL in 96 wells plates at an initial optical density of OD_600_ 0.001 in wells containing various concentrations of ferric citrate or heme. The growth of strains was determined by measuring endpoint OD_600_ on day 18 for ferric citrate and day 30 for heme. The optical density of liquid cultures was measured using a BioTek Synergy plate reader. All experiments were performed with a minimum of three biological replicates.

### Ethidium bromide accumulation assay.

Strains were first grown to log phase in 30 mL of complete 7H9 medium and then filtered through a 5.0-μM filter to obtain a single-cell suspension. Cells were then harvested by low-speed centrifugation at 1,500 × *g* for 10 min and resuspended to a final OD_600_ of 1.0 in uptake buffer (76 mM (NH_4_)_2_SO_4_, 0.5 M KH_2_PO, 1 mM MgSO_4_, 0.4% glucose, and 0.05% Tween 80). For both strains, 100 μL of cells was added in triplicate in a 96-well plate and ethidium bromide was then added to a final concentration of 20 μM. Fluorescence was measured by excitation at 530 nm and emission at 590 nm at 1 min intervals for 20 min.

### Transcriptomic analysis, RNA extraction, and real-time PCR.

Iron-depleted wt *Mtb* was inoculated in triplicates into 60 mL of albumin-free iron-free 7H9 containing either 10 μM FeCi or 10 μM heme at an initial optical density of OD_600_ 0.01. Strains were grown to the midexponential phase to OD_600_ ~1.0. Total RNA was extracted exactly as we did previously (8) and mRNA was isolated using NEBNext rRNA Depletion kit (E7850L). RNA sequencing and bioinformatic analysis were performed at the Microbial Pathogenesis and Genomics Core Center at the University of Oklahoma Health Science Center. Sequence reads were aligned to *Mtb* H37Rv genome (NC_000962). Differential expression analysis was performed with DESeq (21) comparing the heme growth condition to the standard FeCi condition. Three hundred seventeen out of 3,968 genes (8.5%) with nonzero read counts were differentially expressed at least 2-fold with a false discovery rate (FDR) adjusted *P* < 0.01 (181 up in heme, 135 down). To compare expression levels of genes between wt and Δ*sigM*, iron-depleted strains were inoculated at OD_600_ of 1.0 in triplicates into 60 mL of albumin-free iron-free 7H9 containing 10 μM heme and incubated for 48 h. Cells were harvested, and total RNA was extracted. cDNA synthesis was performed using the Bio-Rad iScript cDNA Synthesis kit as per the manufacturer’s protocol. qRT-PCR cycling conditions for relative quantitation of gene expression were performed using Realplex EP Gradient S Real-Time cycler (Eppendorf). Cycle threshold (*C_t_*) data were normalized to *rrs* (*Mtb* 16S rRNA gene), and normalized *C_t_* values (Δ*C_t_*) were transformed to arbitrary gene expression units using the 2^−Δ^*^Ct^*/10^−6^ method as described by Livak and Schmittgen (36).

### Targeted gene deletion in *Mtb*.

To construct mutants, 1,000 bp of left (L) and 1,000 bp right (R) flanking sequences of the target gene were amplified using corresponding primer pairs LF/SpeI-LR/SwaI and RF/PacI-RR/NsiI (Table S3), respectively, and cloned into pML2424 to construct gene deletion vectors (Table S3). The deletion vectors were then transformed into *Mtb*. Transformants were selected at 37°C on 7H10 Hyg and visually validated through the presence of both GFP and RFP fluorescence. Liquid culture of the transformant was then plated on 7H10 Hyg containing 2% sucrose at 40°C for the selection of double crossovers. Putative double crossovers were visually analyzed for the presence of only GFP, and gene deletion was validated by PCR. For excision of the *loxP*-flanked *gfp*^2+^*_m_-hyg* cassette, pML2714 expressing Cre recombinase was transformed into marked mutants and unmarked mutants were selected on 7H10 Kan at 37°C. Putative unmarked mutants were first visually validated through the absence of GFP fluorescence and then through PCR (Fig. S1; Fig. S3) (validation primers [V/F–V/R], Table S3) and loss of growth on hygromycin. All validated mutants were designated an OALnumber (Table S3) for identification.

### Construction of mycobacterial expression and protein purification vectors.

All primers and vectors are described in Table S3. The orf of *eccC4* was cloned into pML1335 vector using corresponding primers to generate the *eccC4* integrative expression vector pOAL102. The ORF of *hs1-M7A* was cloned into pMN016 using corresponding primers to generate the episomal heme sensor expression vector pOAL311. C-terminally HA-tagged integrative expression vectors for *ppe36* (pOAL313), *ppe62* (pOAL314), and *ppe64* (pOAL317) were generated by cloning ORFs into pML1335 using corresponding primers. Integrative expression vectors for *rv0125* (pOAL319), *rv1085c* (pOAL320), and *sigM* (pOAL321) were generated by cloning ORFs into pML2300 using corresponding primers. All mycobacterial expression vectors use the strong psymc promoter for gene expression. All vectors used for protein purification were constructed by cloning ORFs into pET21a+ using corresponding primers.

### Temporal analysis of heme biosensors.

Transformants of pOAL311 (HS1-M7A) in wt and Δ*eccC4* were selected on 7H10 Hyg agar plates. Iron-depleted strains were inoculated at OD_600_ of 1.0 in triplicates into 10 mL of albumin-free iron-free 7H9 containing either 10 μM FeCi or 10 μM heme. Cells were grown in the specific medium, and fluorescence for each biosensor was measured at the specific time points as mentioned in the text (Fig. 3). eGFP fluorescence was measured by excitation/emission at 480 nm/510 nm, and mKATE2 fluorescence was measured by excitation/emission at 580 nm/620 nm. HS1-M7A fluorescence is reported as a ratio of eGFP/mKATE2. For determining biosensor functionality (Fig. 3A), the wt transformant was inoculated in the medium at an initial optical density of OD_600_ 0.01. For comparing biosensor fluorescence between wt and Δ*eccC4* (Fig. 3B), strains were inoculated at a high initial optical density of OD_600_ 1.0 because the *eccC4* strain has a significant growth delay in heme and so that enough cells were available for fluorescence measurements. Optical density and fluorescence were measured using a BioTek Synergy plate reader.

### Subcellular fractionation and surface accessibility assays.

All experiments were performed as we did previously (9). Iron-depleted cells (Table S3) were grown in 120 mL of 7H9C with Hyg to an OD_600_ of 2. Cells were lysed by sonication and soluble and membrane fractions were separated by ultracentrifugation at 100,000 × *g* for 1 h at 4°C. The supernatant (C1) was transferred to a separate tube, and the pellet was resuspended in the same volume of PBS as C1 and designated M1. Both C1 and M1 fractions were centrifuged at 100,000 × *g* for 1 h at 4°C. The supernatant containing the cytosolic fraction was transferred to a new tube and labeled C2, and the membrane pellet fraction was resuspended in the same volume of PBS as that used for C2 and designated M2. HA-tagged proteins in L, M2, and C2 fractions were detected in Western blots using primary mouse anti-HA (Invitrogen; MA527543) antibody. Primary monoclonal mouse antibodies were used for detecting LpqH (BEI; NR-50098) and RNA polymerase (Invitrogen; MA125425). Secondary horseradish peroxidase-conjugated goat anti-mouse (Bio-Rad; 1706516) antibody was in all blots, which were developed using ECL substrate (Bio-Rad; 1705061), and luminescence was visualized using Bio-Rad Chemidoc MP imaging system. Strains expressing HA-tagged proteins were grown to mid-log phase and fixed with 4% paraformaldehyde for 30 min at room temperature. The cells were washed twice with PBS-Tyloxapol (0.02%) and incubated with monoclonal rabbit anti-HA antibody at a 1:1 dilution for 2 h for bacterial surface staining. The cells were then washed three times with PBS-Tyloxapol and then stained with fluorescein isothiocyanate (FITC)-labeled anti-rabbit antibodies at a dilution of 1:100 for 2 h. The cells were again washed three times with PBS-Tyloxapol and analyzed via flow cytometry. Surface-accessible proteins were quantified by measuring fluorescence and displayed as histograms.

### Harvesting culture filtrate and proteomic analysis.

Iron-depleted *Mtb* and *ΔeccC4* were inoculated in five replicates into 30 mL of albumin-free iron-free 7H9 containing either 10 μM FeCi or 10 μM heme at a high initial optical density of OD_600_ 1.0 to ensure enough cells for appropriate comparison of protein levels. FeCi culture supernatants were harvested after 8 days when both strains exhibit exponential growth (Fig. 2C). Heme culture supernatant for both strains was harvested after 20 days when wt is in exponential phase and Δ*eccC4* is still in lag phase (Fig. 2D). OD_600_ of wt and Δ*eccC4* heme cultures were ~4.3 and ~1.6, respectively. Culture supernatant was harvested by centrifugation at 5,000 × *g* for 10 min and then filtered through a 0.2-μm filter. Amicon 3-kDa centrifugal filtration units were thoroughly washed with millipore water, and the filtered supernatant was concentrated >60-fold.

Fifty micrograms of protein samples was digested using the S-trap protocol, as described by the manufacturer (37). For this, samples were adjusted to contain 5% SDS, 5 mM Tris(2-carboxyethyl)phosphine, 50 mM triethylammonium bicarbonate (TEAB), pH 8.5, and the samples were incubated for 30 min at room temperature (RT). Samples were then alkylated by adding iodoacetamide to 10 mM and incubating for 20 min at RT. The alkylated samples were then acidified by adding phosphoric acid to a 1.2% final concentration (C_f_) and diluted further by adding 6 volumes of buffered methanol (90% methanol; 100 mM TEAB, pH 7.1). Samples were centrifuged through the S-trap spin filtration devices (Protifi number C02-micro), and the retentates were washed further with buffered methanol. To each washed retentate, 25 μL of 50 mM triethylammonium bicarbonate pH 8.5 containing 0.8 μg of trypsin/LysC (Promega) was applied, and the digestions were incubated overnight at 37°C. After digestion, peptides were collected via three successive 40-μL elutions first using 40 mM TEAB pH 8.5, then 0.1% aqueous formic acid, and then 50:50:0.1 acetonitrile/water/formic acid, after which the pooled peptide eluates were dried by vacuum centrifugation. Peptides were analyzed by liquid chromatography-tandem mass spectrometry (LC-MS/MS) on a quadrupole-Orbitrap mass spectrometer (Fusion model, Thermo), using a “high/low” data-dependent MS/MS acquisition method. For these analyses, peptides were dissolved in mobile phase A (0.1% aqueous formic acid), and a vented trap configuration was used for injection onto a 75-μm × 50-cm analytical column packed with 2-μm C18 particles (Acclaim PepMap RSLC, number 20330952, Thermo). The column was developed using 80:20:0.1 acetonitrile/water/formic acid as mobile phase B, transitioning linearly from 4% mobile phase B to 32% mobile phase B over a period of 120 min at a uniform 250 nL/min flow rate. Eluting peptides were ionized in a Nanospray Flex ion source (Thermo) using a stainless-steel emitter. Peptide ions were analyzed in the Orbitrap sector at a nominal resolution of 120,000, selected for MS/MS using the quadrupole sector, fragmented by higher-energy collisional dissociation in the ion routing multipole sector, and the fragment ions analyzed in the ion trap sector. The specific instrument settings are provided in Table S2 and are also available through the PRIDE data archive (project PXD036081).

To identify and quantify peptides and infer protein abundances, MaxQuant v2.0.1.0 (89) was used to analyze the raw instrument files. The reference proteome of 3,993 M. tuberculosis H37Rv sequences was downloaded from Uniprot. Searches utilized default settings in MaxQuant, supplemented with the variable modification cyclization of glutamine to pyroglutamate, with the Match Between Runs enabled, and with LFQ and iBAQ quantitations enabled (90). The label-free quantification (LFQ) values of 2,615 proteins (Table S2) were further used to compare proteomes. Reversed decoy proteins identifications and contaminants were filtered out and protein intensities were log_2_ transformed. Data were normalized by subtracting the median. SAM analysis (38) was performed using the volcano plot tool in Perseus (91) using a permutation-corrected FDR of 5% to control for multiple hypotheses testing one-way and an S0 value of 0.1.

### Isolation of inclusion bodies, protein purification, and protein refolding.

Transformants of pOAL301, pOAL306, and pOAL312 in E. coli BL21(DE3) were selected on LB Amp agar plates. All proteins were purified using the same method. Briefly, starter cultures of strains were inoculated into 500 mL of fresh LB medium with ampicillin and grown to an OD_600_ of 0.3. Gene expression was induced with 1 mM IPTG at 18°C for 18 h. After induction of gene expression E. coli, cells were harvested by centrifugation and lysed by sonication in ice-cold base buffer (BB; 50 mM NaPi, 150 mM NaCl, pH 7.4). The cell lysate was clarified by low-speed centrifugation at 1,500 × *g*, 4°C for 10 min, and the supernatant containing whole-cell lysate (Fig. 7A, lane 1) was removed to a fresh tube. To solubilize all membrane proteins, Triton X-100 was added to the lysate at 1% C_f_ and incubated on ice for 20 min. The lysate was then centrifuged at 15,000 × *g*, 4°C for 15 min, and the supernatant (Fig. 7A, lane 2) was removed leaving the inclusion bodies (IB) in the pellet. The IB pellet was washed twice with BB (Fig. 7A, lanes 3 to 4) and then solubilized with BB containing 8 M Urea (BBU) (Fig. 7A, lane 5). Solubilized IB was loaded on to activated nickel resin and bound at room temperature for 2 h. Flow through was collected (Fig. 7A, lane 6), and protein-loaded resin was washed twice with BBU containing 40 mM imidazole (Fig. 7A, lanes 7 to 8) wash buffer. The target protein was eluted with BBU containing 500 mM imidazole (Fig. 7A, lanes 9 to 15). The cleanest elutions as visualized through gel electrophoresis were used for subsequent dialysis and refolding of protein. OPOE or DDM detergent was added to eluted protein to 0.5% or 0.1% C_f_, respectively. Dialysis buffer (DB) was prepared by adding OPOE or DDM to BB to 0.5% or 0.1% C_f_, respectively. A protein sample (1 mL) was loaded into a Thermo Slide-A-Lyzer 3.5-kDa dialysis cassette, and urea was removed by dialysis with 400 mL of DB. DB was replaced at 24 h, and dialysis was performed for a total of 48 h at 4°C with low spin. PPE36, MhuD, and IdeR were all purified as we did previously (9). MspA was selectively purified by heat extraction as described previously (42). Briefly, a 50-mL culture of M. smegmatis mc^2^155 was first grown in 7H9 medium to the midexponential phase. Cells were harvested by centrifugation at 5,000 × *g* for 5 min, washed three times with PBS, and then resuspended in 0.3 mL of PG05 buffer (0.5% Genapol, 100 mM Na_2_HPO_4_/NaH_2_PO_4_, 0.1 mM EDTA, and 150 mM NaCl, pH 6.5). Protein was first extracted by incubating cells for 30 min at 100°C, the sample was then cooled on ice and centrifuged to remove debris, and the supernatant containing MspA was used for SDS-PAGE and bilayer analysis.

### Absorption and surface plasmon resonance spectroscopy for detecting heme binding.

Fresh solutions of hemin were prepared in Tris buffer. An equimolar amount of heme was added to 10 μM protein and incubated a room temperature for 5 min. For difference absorption spectroscopy, heme binding was monitored using a Bio-Tek Synergy HT plate reader by subtracting the free heme spectra from the protein incubated heme spectra. Surface plasmon resonance (SPR) experiments were performed at University of Alabama at Birmingham SPR core facility exactly as we did before (9).

### Lipid bilayer experiments, recording, and data analysis.

Bilayer experiments were performed using very similar instrumentation and methods as described by Zakharian and Reusch (92). Synthetic diphytanoyl phosphatidylcholine (DphPC; Avanti Polar Lipids, Birmingham, AL) was used to form planar lipid bilayers. Lipids were solubilized in *n*-Decane at 20 mg/mL, and a glass capillary tube was used to paint a bilayer in an aperture of 200 μM diameter in a Delrin cup (Warner Instruments, Hamden, CT). The bilayer was painted between an aqueous solution of 1 M KCl, 10 mM HEPES, pH 7.1, and capacitance was registered in the range of 66 to 100 pF. Approximately ~40 ng of purified OPOE refolded protein in 1 to 2 μL volume was added to the *cis* compartment and channel-forming activity was recorded at 30-mV applied potential. The current trace was recorded with a patch-clamp amplifier (BC-535 Bilayer Clamp, Warner Instruments). The *trans* and *cis* solutions were connected to the head stage point with Ag-AgCl electrodes. Currents were low-pass filtered at 10 kHz and then digitized through an analog-to-digital converter (Digidata 1550B; Molecular Devices, San Jose, CA). Data filtering was done at 100 Hz through an 8-pole Bessel Filter (Lpf-8; Warner Instruments) and digitized at 1 kHz using pClamp11 software (Molecular Devices). Single-channel conductance events were identified automatically using Clampfit11 from 5 independent membrane recordings.

### Data availability.

All source data files are provided and/or publicly available and are also available to anyone upon request. RNAseq data files are available through the NCBI Sequence Read Archive (BioProject ID PRJNA868362). Mass spectrometry data files are available through the PRIDE Data Archive (PXD036081). Lipid bilayer data acquisition files (axon binary file) require pClamp software, which to the best of our knowledge is only available for purchase through Molecular Devices. All requests should be addressed to Avishek Mitra.

## References

[1] World Health Organization. 2021. Global tuberculosis report 2021. World Health Organization, Geneva, Switzerland.

[2] Skaar EP. 2010. The battle for iron between bacterial pathogens and their vertebrate hosts. PLoS Pathog 6:e1000949. doi:10.1371/journal.ppat.1000949. 20711357 PMC2920840

[3] Cassat JE, Skaar EP. 2013. Iron in infection and immunity. Cell Host Microbe 13:509–519. doi:10.1016/j.chom.2013.04.010. 23684303 PMC3676888

[4] Skaar EP, Raffatellu M. 2015. Metals in infectious diseases and nutritional immunity. Metallomics 7:926–928. doi:10.1039/c5mt90021b. 26017093

[5] White AJ, Snow GA. 1969. Isolation of mycobactinss from various mycobacteria. The properties of mycobactin S and H. Biochem J 111:785–792. doi:10.1042/bj1110785. 5783478 PMC1187608

[6] Snow GA. 1969. Metal complexes of mycobactin P and of desferrisideramines. Biochem J 115:199–205. doi:10.1042/bj1150199. 5378379 PMC1185090

[7] Runyen-Janecky LJ. 2013. Role and regulation of heme iron acquisition in gram-negative pathogens. Front Cell Infect Microbiol 3:55. doi:10.3389/fcimb.2013.00055. 24116354 PMC3792355

[8] Mitra A, Ko YH, Cingolani G, Niederweis M. 2019. Heme and hemoglobin utilization by Mycobacterium tuberculosis. Nat Commun 10:4260. doi:10.1038/s41467-019-12109-5. 31534126 PMC6751184

[9] Mitra A, Speer A, Lin K, Ehrt S, Niederweis M. 2017. PPE surface proteins are required for heme utilization by Mycobacterium tuberculosis. mBio 8:e01720-16. doi:10.1128/mBio.01720-16. 28119467 PMC5263243

[10] Jones CM, Niederweis M. 2011. *Mycobacterium tuberculosis* can utilize heme as an iron source. J Bacteriol 193:1767–1770. doi:10.1128/JB.01312-10. 21296960 PMC3067660

[11] Tullius MV, Harmston CA, Owens CP, Chim N, Morse RP, McMath LM, Iniguez A, Kimmey JM, Sawaya MR, Whitelegge JP, Horwitz MA, Goulding CW. 2011. Discovery and characterization of a unique mycobacterial heme acquisition system. Proc Natl Acad Sci USA 108:5051–5056. doi:10.1073/pnas.1009516108. 21383189 PMC3064333

[12] Huang W, Wilks A. 2017. Extracellular heme uptake and the challenge of bacterial cell membranes. Annu Rev Biochem 86:799–823. doi:10.1146/annurev-biochem-060815-014214. 28426241

[13] Tullius MV, Nava S, Horwitz MA. 2019. PPE37 is essential for mycobacterium tuberculosis heme-iron acquisition (HIA), and a defective PPE37 in Mycobacterium bovis BCG prevents HIA. Infect Immun 87:e00540-18. doi:10.1128/IAI.00540-18. PMC6346139 30455201

[14] Adams PA, Berman MC. 1980. Kinetics and mechanism of the interaction between human serum albumin and monomeric haemin. Biochem J 191:95–102. doi:10.1042/bj1910095. 7470101 PMC1162185

[15] Pinsky M, Roy U, Moshe S, Weissman Z, Kornitzer D. 2020. Human serum albumin facilitates heme-iron utilization by fungi. mBio 11:e00607-20. doi:10.1128/mBio.00607-20. 32317324 PMC7175094

[16] Groschel MI, Sayes F, Simeone R, Majlessi L, Brosch R. 2016. ESX secretion systems: mycobacterial evolution to counter host immunity. Nat Rev Microbiol 14:677–691. doi:10.1038/nrmicro.2016.131. 27665717

[17] Vaziri F, Brosch R. 2019. ESX/Type VII secretion systems–an important way out for mycobacterial proteins. Microbiol Spectr 7:7.4.10. doi:10.1128/microbiolspec.PSIB-0029-2019. PMC10957191 31298207

[18] Fishbein S, van Wyk N, Warren RM, Sampson SL. 2015. Phylogeny to function: PE/PPE protein evolution and impact on Mycobacterium tuberculosis pathogenicity. Mol Microbiol 96:901–916. doi:10.1111/mmi.12981. 25727695

[19] Sampson SL. 2011. Mycobacterial PE/PPE proteins at the host-pathogen interface. Clin Dev Immunol 2011:497203. doi:10.1155/2011/497203. 21318182 PMC3034920

[20] Rodriguez GM, Voskuil MI, Gold B, Schoolnik GK, Smith I. 2002. ideR, An essential gene in mycobacterium tuberculosis: role of IdeR in iron-dependent gene expression, iron metabolism, and oxidative stress response. Infect Immun 70:3371–3381. doi:10.1128/IAI.70.7.3371-3381.2002. 12065475 PMC128082

[21] Anders S, Huber W. 2010. Differential expression analysis for sequence count data. Genome Biol 11:R106. doi:10.1186/gb-2010-11-10-r106. 20979621 PMC3218662

[22] Quadri LE, Sello J, Keating TA, Weinreb PH, Walsh CT. 1998. Identification of a Mycobacterium tuberculosis gene cluster encoding the biosynthetic enzymes for assembly of the virulence-conferring siderophore mycobactin. Chem Biol 5:631–645. doi:10.1016/s1074-5521(98)90291-5. 9831524

[23] Jones CM, Wells RM, Madduri AV, Renfrow MB, Ratledge C, Moody DB, Niederweis M. 2014. Self-poisoning of Mycobacterium tuberculosis by interrupting siderophore recycling. Proc Natl Acad Sci USA 111:1945–1950. doi:10.1073/pnas.1311402111. 24497493 PMC3918798

[24] Wells RM, Jones CM, Xi Z, Speer A, Danilchanka O, Doornbos KS, Sun P, Wu F, Tian C, Niederweis M. 2013. Discovery of a siderophore export system essential for virulence of Mycobacterium tuberculosis. PLoS Pathog 9:e1003120. doi:10.1371/journal.ppat.1003120. 23431276 PMC3561183

[25] Arnold FM, Weber MS, Gonda I, Gallenito MJ, Adenau S, Egloff P, Zimmermann I, Hutter CAJ, Hurlimann LM, Peters EE, Piel J, Meloni G, Medalia O, Seeger MA. 2020. The ABC exporter IrtAB imports and reduces mycobacterial siderophores. Nature 580:413–417. doi:10.1038/s41586-020-2136-9. 32296173 PMC7170716

[26] Ryndak MB, Wang S, Smith I, Rodriguez GM. 2010. The Mycobacterium tuberculosis high-affinity iron importer, IrtA, contains an FAD-binding domain. J Bacteriol 192:861–869. doi:10.1128/JB.00223-09. 19948799 PMC2812465

[27] Rodriguez GM, Smith I. 2006. Identification of an ABC transporter required for iron acquisition and virulence in Mycobacterium tuberculosis. J Bacteriol 188:424–430. doi:10.1128/JB.188.2.424-430.2006. 16385031 PMC1347291

[28] Hanna DA, Martinez-Guzman O, Reddi AR. 2017. Heme gazing: illuminating eukaryotic heme trafficking, dynamics, and signaling with fluorescent heme sensors. Biochemistry 56:1815–1823. doi:10.1021/acs.biochem.7b00007. 28316240 PMC5629415

[29] Hanna DA, Harvey RM, Martinez-Guzman O, Yuan X, Chandrasekharan B, Raju G, Outten FW, Hamza I, Reddi AR. 2016. Heme dynamics and trafficking factors revealed by genetically encoded fluorescent heme sensors. Proc Natl Acad Sci USA 113:7539–7544. doi:10.1073/pnas.1523802113. 27247412 PMC4941510

[30] Donegan RK, Fu Y, Copeland J, Idga S, Brown G, Hale OF, Mitra A, Yang H, Dailey HA, Niederweis M, Jain P, Reddi AR. 2022. Exogenously scavenged and endogenously synthesized heme are differentially utilized by Mycobacterium tuberculosis. Microbiol Spectr 10:e0360422. doi:10.1128/spectrum.03604-22. 36169423 PMC9604157

[31] Kaps I, Ehrt S, Seeber S, Schnappinger D, Martin C, Riley LW, Niederweis M. 2001. Energy transfer between fluorescent proteins using a co-expression system in Mycobacterium smegmatis. Gene 278:115–124. doi:10.1016/s0378-1119(01)00712-0. 11707328

[32] Kapopoulou A, Lew JM, Cole ST. 2011. The MycoBrowser portal: a comprehensive and manually annotated resource for mycobacterial genomes. Tuberculosis (Edinb) 91:8–13. doi:10.1016/j.tube.2010.09.006. 20980200

[33] Arraiz N, Salazar L, Lopez G, Rodriguez R, Casart Y, Takiff H. 2001. Characterization of the expression and function of SigM an ECF sigma factor in mycobacteria. Acta Cient Venez 52(Suppl 1):40–41. 11899704

[34] Clark RR, Judd J, Lasek-Nesselquist E, Montgomery SA, Hoffmann JG, Derbyshire KM, Gray TA. 2018. Direct cell-cell contact activates SigM to express the ESX-4 secretion system in Mycobacterium smegmatis. Proc Natl Acad Sci USA 115:E6595–E6603. doi:10.1073/pnas.1804227115. 29941598 PMC6048512

[35] Chim N, Iniguez A, Nguyen TQ, Goulding CW. 2010. Unusual diheme conformation of the heme-degrading protein from *Mycobacterium tuberculosis*. J Mol Biol 395:595–608. doi:10.1016/j.jmb.2009.11.025. 19917297 PMC2859679

[36] Livak KJ, Schmittgen TD. 2001. Analysis of relative gene expression data using real-time quantitative PCR and the 2(-delta delta C(T)) method. Methods 25:402–408. doi:10.1006/meth.2001.1262. 11846609

[37] HaileMariam M, Eguez RV, Singh H, Bekele S, Ameni G, Pieper R, Yu Y. 2018. S-Trap, an Ultrafast sample-preparation approach for shotgun proteomics. J Proteome Res 17:2917–2924. doi:10.1021/acs.jproteome.8b00505. 30114372

[38] Tusher VG, Tibshirani R, Chu G. 2001. Significance analysis of microarrays applied to the ionizing radiation response. Proc Natl Acad Sci USA 98:5116–5121. doi:10.1073/pnas.091062498. 11309499 PMC33173

[39] Korycka-Machała M, Pawełczyk J, Borówka P, Dziadek B, Brzostek A, Kawka M, Bekier A, Rykowski S, Olejniczak AB, Strapagiel D, Witczak Z, Dziadek J. 2020. PPE51 is involved in the uptake of disaccharides by Mycobacterium tuberculosis. Cells 9:603. doi:10.3390/cells9030603. 32138343 PMC7140425

[40] Wang Q, Boshoff HIM, Harrison JR, Ray PC, Green SR, Wyatt PG, Barry CE, III. 2020. PE/PPE proteins mediate nutrient transport across the outer membrane of Mycobacterium tuberculosis. Science 367:1147–1151. doi:10.1126/science.aav5912. 32139546 PMC11036889

[41] Faller M, Niederweis M, Schulz GE. 2004. The structure of a mycobacterial outer-membrane channel. Science 303:1189–1192. doi:10.1126/science.1094114. 14976314

[42] Heinz C, Niederweis M. 2000. Selective extraction and purification of a mycobacterial outer membrane protein. Anal Biochem 285:113–120. doi:10.1006/abio.2000.4728. 10998270

[43] Pavlenok M, Niederweis M. 2016. Hetero-oligomeric MspA pores in Mycobacterium smegmatis. FEMS Microbiol Lett 363:fnw046 doi:10.1093/femsle/fnw046. 26912121 PMC5975246

[44] Gray TA, Clark RR, Boucher N, Lapierre P, Smith C, Derbyshire KM. 2016. Intercellular communication and conjugation are mediated by ESX secretion systems in mycobacteria. Science 354:347–350. doi:10.1126/science.aag0828. 27846571 PMC8324006

[45] Laencina L, Dubois V, Le Moigne V, Viljoen A, Majlessi L, Pritchard J, Bernut A, Piel L, Roux AL, Gaillard JL, Lombard B, Loew D, Rubin EJ, Brosch R, Kremer L, Herrmann JL, Girard-Misguich F. 2018. Identification of genes required for Mycobacterium abscessus growth in vivo with a prominent role of the ESX-4 locus. Proc Natl Acad Sci USA 115:E1002–E1011. doi:10.1073/pnas.1713195115. 29343644 PMC5798338

[46] Izquierdo Lafuente B, Ummels R, Kuijl C, Bitter W, Speer A. 2021. Mycobacterium tuberculosis toxin CpnT Is an ESX-5 substrate and requires three type VII secretion systems for intracellular secretion. mBio 12:e02983-20. doi:10.1128/mBio.02983-20. 33653883 PMC8092274

[47] Wang Y, Tang Y, Lin C, Zhang J, Mai J, Jiang J, Gao X, Li Y, Zhao G, Zhang L, Liu J. 2022. Crosstalk between the ancestral type VII secretion system ESX-4 and other T7SS in Mycobacterium marinum. iScience 25:103585. doi:10.1016/j.isci.2021.103585. 35005535 PMC8718981

[48] Pajuelo D, Tak U, Zhang L, Danilchanka O, Tischler AD, Niederweis M. 2021. Toxin secretion and trafficking by Mycobacterium tuberculosis. Nat Commun 12:6592. doi:10.1038/s41467-021-26925-1. 34782620 PMC8593097

[49] Siegrist MS, Steigedal M, Ahmad R, Mehra A, Dragset MS, Schuster BM, Philips JA, Carr SA, Rubin EJ. 2014. Mycobacterial Esx-3 requires multiple components for iron acquisition. mBio 5:e01073-14–e01014. doi:10.1128/mBio.01073-14. 24803520 PMC4010830

[50] Serafini A, Pisu D, Palu G, Rodriguez GM, Manganelli R. 2013. The ESX-3 secretion system is necessary for iron and zinc homeostasis in Mycobacterium tuberculosis. PLoS One 8:e78351. doi:10.1371/journal.pone.0078351. 24155985 PMC3796483

[51] Serafini A, Boldrin F, Palu G, Manganelli R. 2009. Characterization of a Mycobacterium tuberculosis ESX-3 conditional mutant: essentiality and rescue by iron and zinc. J Bacteriol 191:6340–6344. doi:10.1128/JB.00756-09. 19684129 PMC2753049

[52] Siegrist MS, Unnikrishnan M, McConnell MJ, Borowsky M, Cheng TY, Siddiqi N, Fortune SM, Moody DB, Rubin EJ. 2009. Mycobacterial Esx-3 is required for mycobactin-mediated iron acquisition. Proc Natl Acad Sci USA 106:18792–18797. doi:10.1073/pnas.0900589106. 19846780 PMC2774023

[53] Kurthkoti K, Amin H, Marakalala MJ, Ghanny S, Subbian S, Sakatos A, Livny J, Fortune SM, Berney M, Rodriguez GM. 2017. The capacity of mycobacterium tuberculosis to survive iron starvation might enable it to persist in iron-deprived microenvironments of human granulomas. mBio 8:e01092-17. doi:10.1128/mBio.01092-17. 28811344 PMC5559634

[54] Rivera-Calzada A, Famelis N, Llorca O, Geibel S. 2021. Type VII secretion systems: structure, functions and transport models. Nat Rev Microbiol 19:567–584. doi:10.1038/s41579-021-00560-5. 34040228

[55] Daleke MH, Ummels R, Bawono P, Heringa J, Vandenbroucke-Grauls CM, Luirink J, Bitter W. 2012. General secretion signal for the mycobacterial type VII secretion pathway. Proc Natl Acad Sci USA 109:11342–11347. doi:10.1073/pnas.1119453109. 22733768 PMC3396530

[56] Poulsen C, Panjikar S, Holton SJ, Wilmanns M, Song YH. 2014. WXG100 Protein Superfamily consists of three subfamilies and exhibits an alpha-Helical C-terminal conserved residue pattern. PLoS One 9:e89313. doi:10.1371/journal.pone.0089313. 24586681 PMC3935865

[57] Agarwal N, Woolwine SC, Tyagi S, Bishai WR. 2007. Characterization of the *Mycobacterium tuberculosis* sigma factor SigM by assessment of virulence and identification of SigM-dependent genes. Infect Immun 75:452–461. doi:10.1128/IAI.01395-06. 17088352 PMC1828396

[58] Raman S, Puyang X, Cheng TY, Young DC, Moody DB, Husson RN. 2006. Mycobacterium tuberculosis SigM positively regulates Esx secreted protein and nonribosomal peptide synthetase genes and down regulates virulence-associated surface lipid synthesis. J Bacteriol 188:8460–8468. doi:10.1128/JB.01212-06. 17028284 PMC1698216

[59] Lagune M, Le Moigne V, Johansen MD, Vasquez Sotomayor F, Daher W, Petit C, Cosentino G, Paulowski L, Gutsmann T, Wilmanns M, Maurer FP, Herrmann JL, Girard-Misguich F, Kremer L. 2022. The ESX-4 substrates, EsxU and EsxT, modulate Mycobacterium abscessus fitness. PLoS Pathog 18:e1010771. doi:10.1371/journal.ppat.1010771. 35960766 PMC9401124

[60] Williamson ZA, Chaton CT, Ciocca WA, Korotkova N, Korotkov KV. 2020. PE5-PPE4-EspG3 heterotrimer structure from mycobacterial ESX-3 secretion system gives insight into cognate substrate recognition by ESX systems. J Biol Chem 295:12706–12715. doi:10.1074/jbc.RA120.012698. 32675282 PMC7476729

[61] Korotkova N, Freire D, Phan TH, Ummels R, Creekmore CC, Evans TJ, Wilmanns M, Bitter W, Parret AH, Houben EN, Korotkov KV. 2014. Structure of the *Mycobacterium tuberculosis* type VII secretion system chaperone EspG5 in complex with PE25-PPE41 dimer. Mol Microbiol 94:367–382. doi:10.1111/mmi.12770. 25155747 PMC4192059

[62] Newton-Foot M, Gey van Pittius NC. 2013. Understanding the evolution and function of the mycobacterial type VII ESX secretion systems (T7SSs) and their substrates. PhD disseration. Stellenbosch University, Stellenbosch, South Africa. https://www.google.com/url?sa=t&rct=j&q=&esrc=s&source=web&cd=&cad=rja&uact=8&ved=2ahUKEwiHxfTyuLz5AhWUlmoFHUpPAMMQFnoECAcQAQ&url=https%3A%2F%2Fscholar.sun.ac.za%2Fbitstream%2Fhandle%2F10019.1%2F79805%2Fnewtonfoot_understanding_2013.pdf%3Fsequence%3D2%26isAllowed%3Dy&usg=AOvVaw17h6P-TGdrilxht4Pjkbqm.

[63] Patel S. 2017. A critical review on serine protease: key immune manipulator and pathology mediator. Allergol Immunopathol (Madr) 45:579–591. doi:10.1016/j.aller.2016.10.011. 28236540 PMC7126602

[64] Otto BR, Sijbrandi R, Luirink J, Oudega B, Heddle JG, Mizutani K, Park SY, Tame JR. 2005. Crystal structure of hemoglobin protease, a heme binding autotransporter protein from pathogenic Escherichia coli. J Biol Chem 280:17339–17345. doi:10.1074/jbc.M412885200. 15728184

[65] Otto BR, van Dooren SJ, Dozois CM, Luirink J, Oudega B. 2002. Escherichia coli hemoglobin protease autotransporter contributes to synergistic abscess formation and heme-dependent growth of Bacteroides fragilis. Infect Immun 70:5–10. doi:10.1128/IAI.70.1.5-10.2002. 11748157 PMC127594

[66] Otto BR, van Dooren SJ, Nuijens JH, Luirink J, Oudega B. 1998. Characterization of a hemoglobin protease secreted by the pathogenic Escherichia coli strain EB1. J Exp Med 188:1091–1103. doi:10.1084/jem.188.6.1091. 9743528 PMC2212542

[67] Drago-Serrano ME, Parra SG, Manjarrez-Hernandez HA. 2006. EspC, an autotransporter protein secreted by enteropathogenic Escherichia coli (EPEC), displays protease activity on human hemoglobin. FEMS Microbiol Lett 265:35–40. doi:10.1111/j.1574-6968.2006.00463.x. 17107418

[68] Grijpstra J, Arenas J, Rutten L, Tommassen J. 2013. Autotransporter secretion: varying on a theme. Res Microbiol 164:562–582. doi:10.1016/j.resmic.2013.03.010. 23567321

[69] Desvaux M, Parham NJ, Henderson IR. 2004. The autotransporter secretion system. Res Microbiol 155:53–60. doi:10.1016/j.resmic.2003.10.002. 14990256

[70] Jacob-Dubuisson F, Fernandez R, Coutte L. 2004. Protein secretion through autotransporter and two-partner pathways. Biochim Biophys Acta 1694:235–257. doi:10.1016/j.bbamcr.2004.03.008. 15546669

[71] van Dooren SJ, Tame JR, Luirink J, Oudega B, Otto BR. 2001. Purification of the autotransporter protein Hbp of Escherichia coli. FEMS Microbiol Lett 205:147–150. doi:10.1111/j.1574-6968.2001.tb10938.x. 11728729

[72] Richard KL, Kelley BR, Johnson JG. 2019. Heme uptake and utilization by gram-negative bacterial pathogens. Front Cell Infect Microbiol 9:81. doi:10.3389/fcimb.2019.00081. 30984629 PMC6449446

[73] Parker MW, Postma JP, Pattus F, Tucker AD, Tsernoglou D. 1992. Refined structure of the pore-forming domain of colicin A at 2.4 A resolution. J Mol Biol 224:639–657. doi:10.1016/0022-2836(92)90550-4. 1373773

[74] Parker MW, Pattus F, Tucker AD, Tsernoglou D. 1989. Structure of the membrane-pore-forming fragment of colicin A. Nature 337:93–96. doi:10.1038/337093a0. 2909895

[75] Dal Peraro M, van der Goot FG. 2016. Pore-forming toxins: ancient, but never really out of fashion. Nat Rev Microbiol 14:77–92. doi:10.1038/nrmicro.2015.3. 26639780

[76] Prados-Rosales R, Weinrick BC, Pique DG, Jacobs WR, Jr, Casadevall A, Rodriguez GM. 2014. Role for *Mycobacterium tuberculosis* membrane vesicles in iron acquisition. J Bacteriol 196:1250–1256. doi:10.1128/JB.01090-13. 24415729 PMC3957709

[77] Layre E. 2020. Trafficking of Mycobacterium tuberculosis envelope components and release within extracellular vesicles: host-pathogen interactions beyond the wall. Front Immunol 11:1230. doi:10.3389/fimmu.2020.01230. 32765485 PMC7378356

[78] Chiplunkar SS, Silva CA, Bermudez LE, Danelishvili L. 2019. Characterization of membrane vesicles released by Mycobacterium avium in response to environment mimicking the macrophage phagosome. Future Microbiol 14:293–313. doi:10.2217/fmb-2018-0249. 30757918 PMC6479280

[79] Prados-Rosales R, Baena A, Martinez LR, Luque-Garcia J, Kalscheuer R, Veeraraghavan U, Camara C, Nosanchuk JD, Besra GS, Chen B, Jimenez J, Glatman-Freedman A, Jacobs WR, Jr, Porcelli SA, Casadevall A. 2011. Mycobacteria release active membrane vesicles that modulate immune responses in a TLR2-dependent manner in mice. J Clin Invest 121:1471–1483. doi:10.1172/JCI44261. 21364279 PMC3069770

[80] Lee J, Kim SH, Choi DS, Lee JS, Kim DK, Go G, Park SM, Kim SH, Shin JH, Chang CL, Gho YS. 2015. Proteomic analysis of extracellular vesicles derived from Mycobacterium tuberculosis. Proteomics 15:3331–3337. doi:10.1002/pmic.201500037. 26201501

[81] Siroy A, Mailaender C, Harder D, Koerber S, Wolschendorf F, Danilchanka O, Wang Y, Heinz C, Niederweis M. 2008. Rv1698 of *Mycobacterium tuberculosis* represents a new class of channel-forming outer membrane proteins. J Biol Chem 283:17827–17837. doi:10.1074/jbc.M800866200. 18434314 PMC2440620

[82] Danilchanka O, Sun J, Pavlenok M, Maueroder C, Speer A, Siroy A, Marrero J, Trujillo C, Mayhew DL, Doornbos KS, Munoz LE, Herrmann M, Ehrt S, Berens C, Niederweis M. 2014. An outer membrane channel protein of Mycobacterium tuberculosis with exotoxin activity. Proc Natl Acad Sci USA 111:6750–6755. doi:10.1073/pnas.1400136111. 24753609 PMC4020113

[83] Arora A, Rinehart D, Szabo G, Tamm LK. 2000. Refolded outer membrane protein A of Escherichia coli forms ion channels with two conductance states in planar lipid bilayers. J Biol Chem 275:1594–1600. doi:10.1074/jbc.275.3.1594. 10636850

[84] Prilipov A, Phale PS, Koebnik R, Widmer C, Rosenbusch JP. 1998. Identification and characterization of two quiescent porin genes, *nmpC* and *ompN*, in *Escherichia coli* BE. J Bacteriol 180:3388–3392. doi:10.1128/JB.180.13.3388-3392.1998. 9642192 PMC107294

[85] Conlan S, Zhang Y, Cheley S, Bayley H. 2000. Biochemical and biophysical characterization of OmpG: a monomeric porin. Biochemistry 39:11845–11854. doi:10.1021/bi001065h. 11009596

[86] Dahan D, Vachon V, Laprade R, Coulton JW. 1994. Voltage gating of porins from Haemophilus influenzae type b. Biochim Biophys Acta 1189:204–211. doi:10.1016/0005-2736(94)90067-1. 8292626

[87] Dulberger CL, Rubin EJ, Boutte CC. 2020. The mycobacterial cell envelope–a moving target. Nat Rev Microbiol 18:47–59. doi:10.1038/s41579-019-0273-7. 31728063

[88] Ekiert DC, Cox JS. 2014. Structure of a PE-PPE-EspG complex from *Mycobacterium tuberculosis* reveals molecular specificity of ESX protein secretion. Proc Natl Acad Sci USA 111:14758–14763. doi:10.1073/pnas.1409345111. 25275011 PMC4205667

[89] Cox J, Mann M. 2008. MaxQuant enables high peptide identification rates, individualized p.p.b.-range mass accuracies and proteome-wide protein quantification. Nat Biotechnol 26:1367–1372. doi:10.1038/nbt.1511. 19029910

[90] Cox J, Hein MY, Luber CA, Paron I, Nagaraj N, Mann M. 2014. Accurate proteome-wide label-free quantification by delayed normalization and maximal peptide ratio extraction, termed MaxLFQ. Mol Cell Proteomics 13:2513–2526. doi:10.1074/mcp.M113.031591. 24942700 PMC4159666

[91] Tyanova S, Temu T, Sinitcyn P, Carlson A, Hein MY, Geiger T, Mann M, Cox J. 2016. The Perseus computational platform for comprehensive analysis of (prote)omics data. Nat Methods 13:731–740. doi:10.1038/nmeth.3901. 27348712

[92] Zakharian E, Reusch RN. 2006. Pore characteristics of nontypeable Haemophilus influenzae outer membrane protein P5 in planar lipid bilayers. Biophys J 91:3242–3248. doi:10.1529/biophysj.106.088781. 16905616 PMC1614495

