# Supplementary figures and images for: Role of the Mycobacterium tuberculosis ESX-4 Secretion System in Heme Iron Utilization and Pore Formation by PPE Proteins
Source: mSphere. 2023 Feb 7;8(2):e00573-22. doi: 10.1128/msphere.00573-22 (PMC10117145; doi:10.1128/msphere.00573-22)

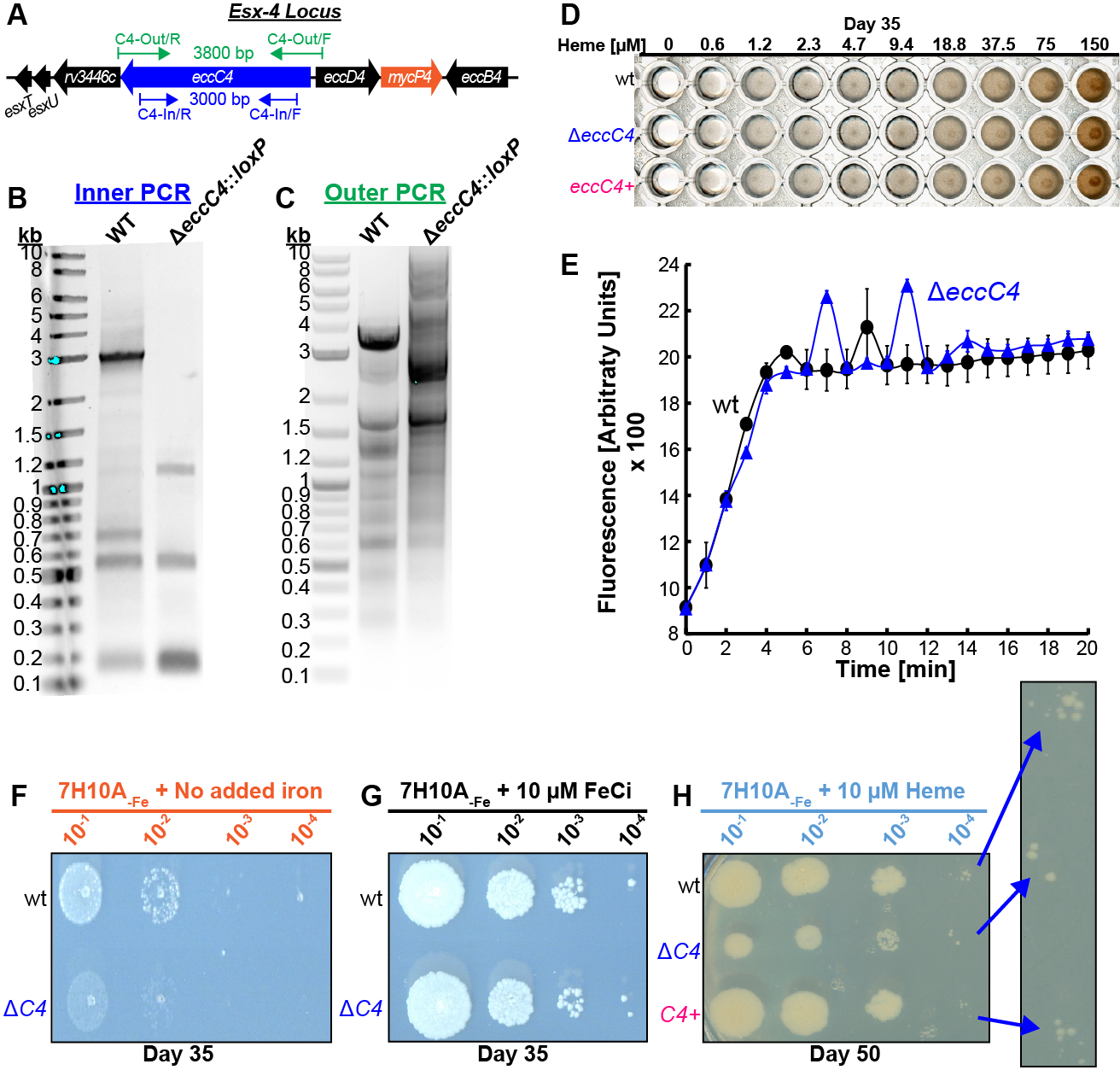

Supplement: FIG S1 [file msphere.00573-22-s0001.tif]

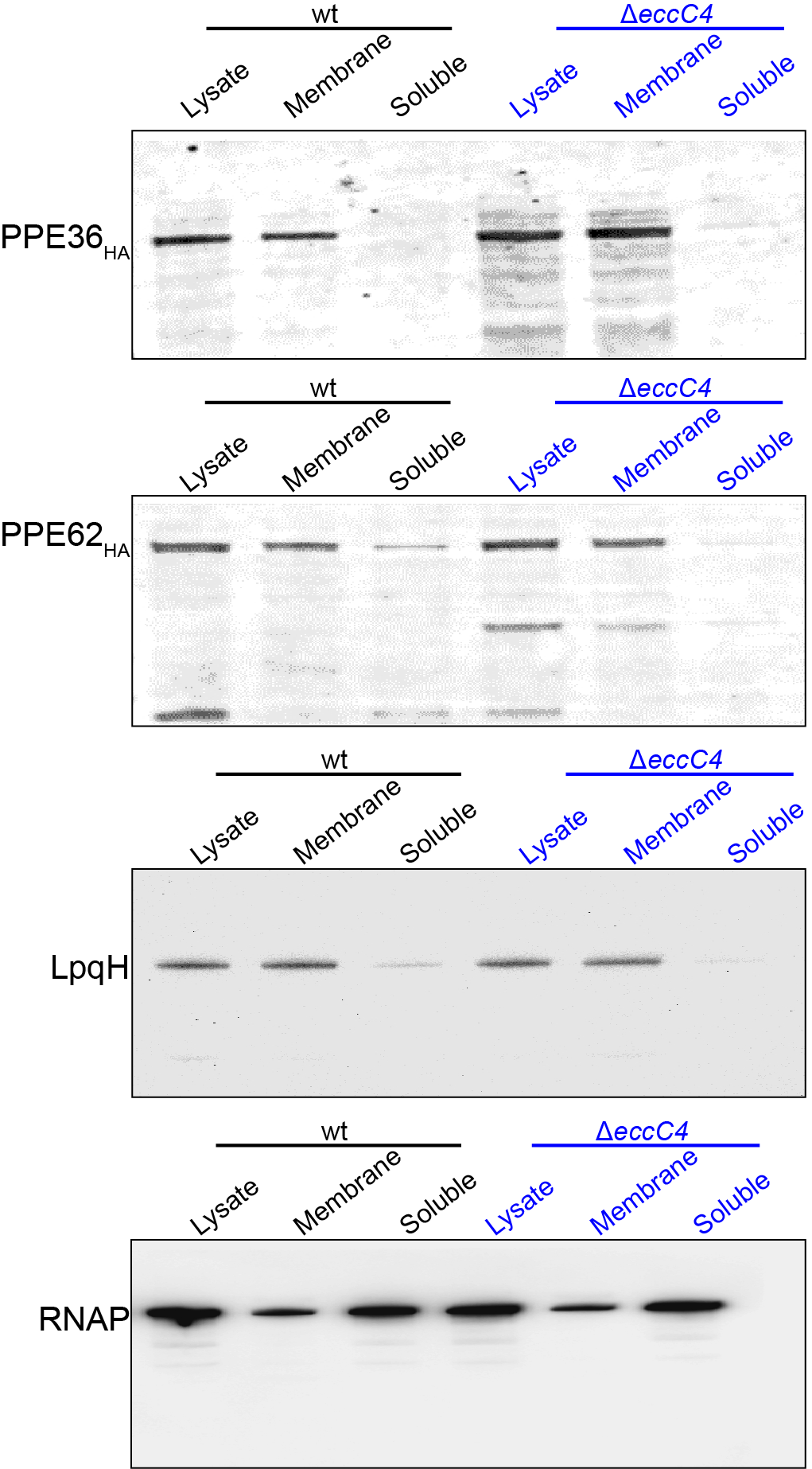

Supplement: FIG S2 [file msphere.00573-22-s0002.tif]

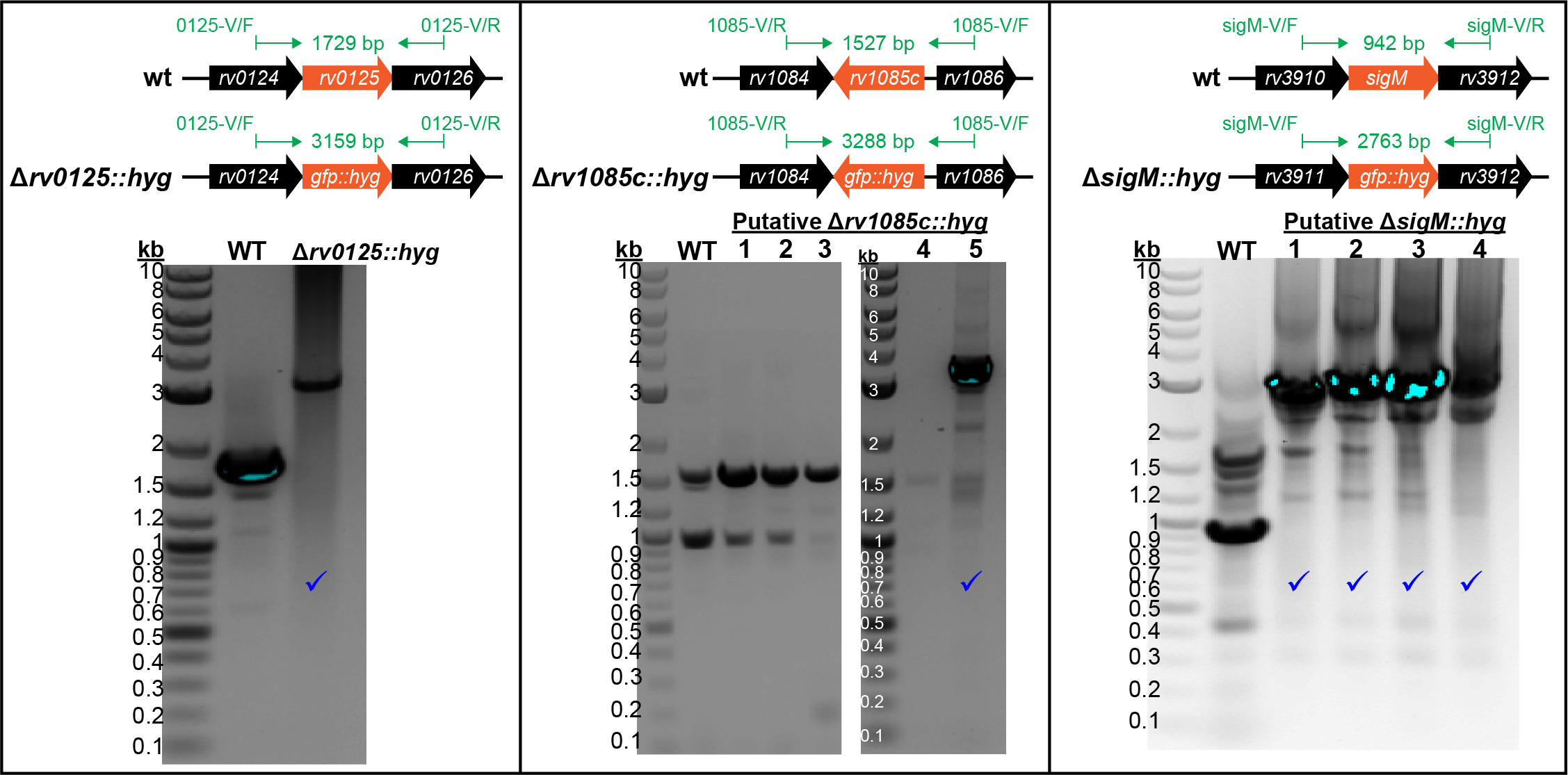

Supplement: FIG S3 [file msphere.00573-22-s0003.tif]

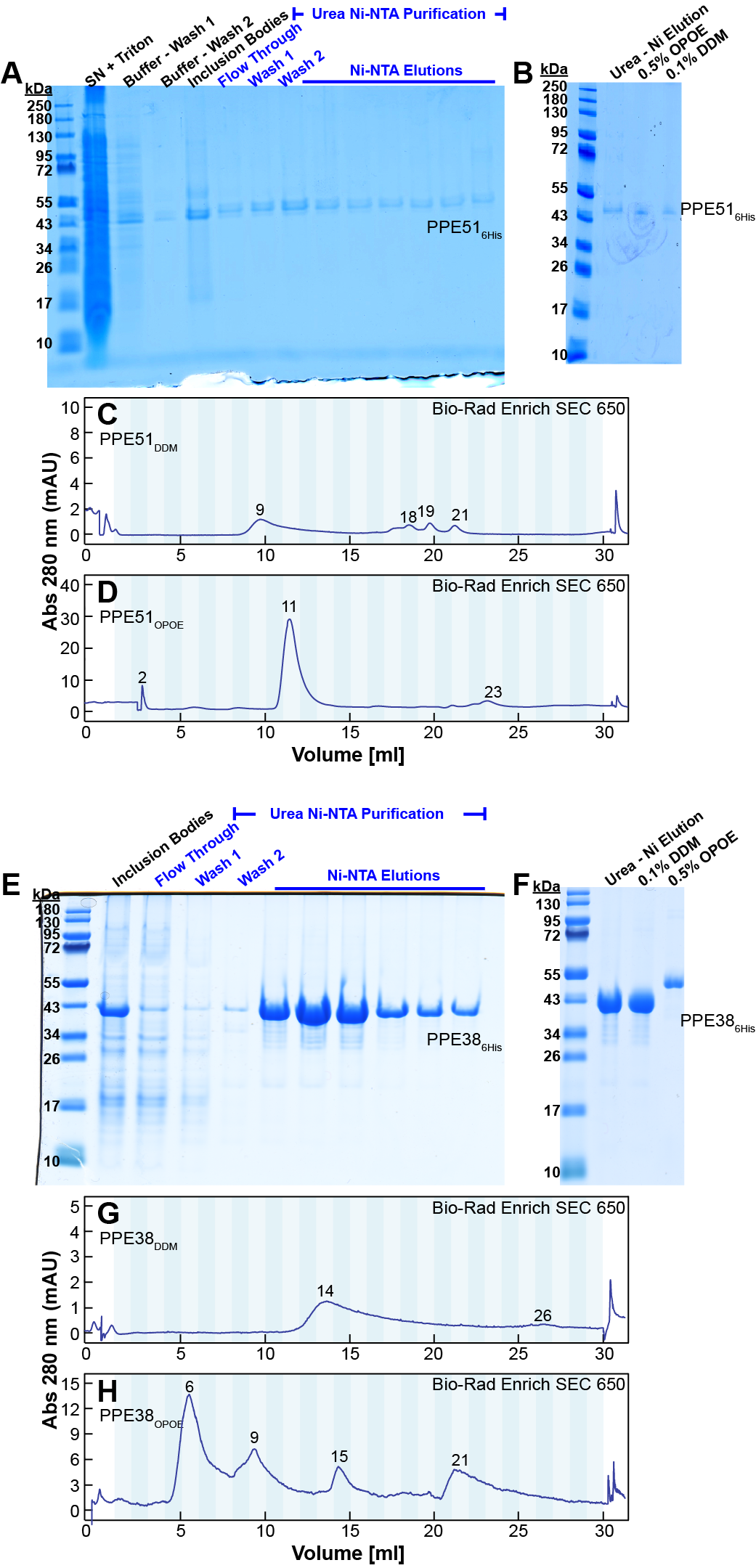

Supplement: FIG S4 [file msphere.00573-22-s0004.tif]

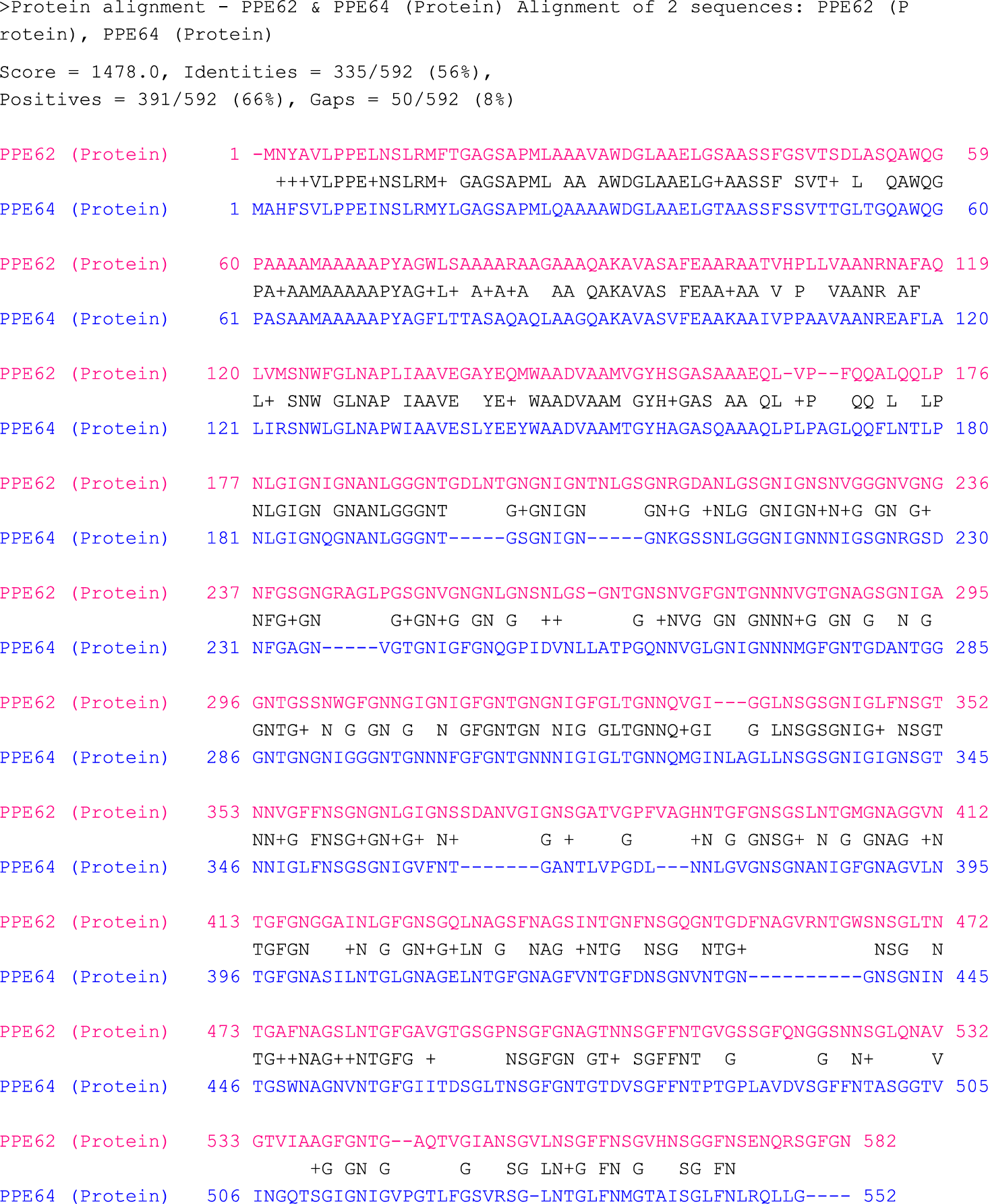

Supplement: FIG S6 [file msphere.00573-22-s0006.tif]

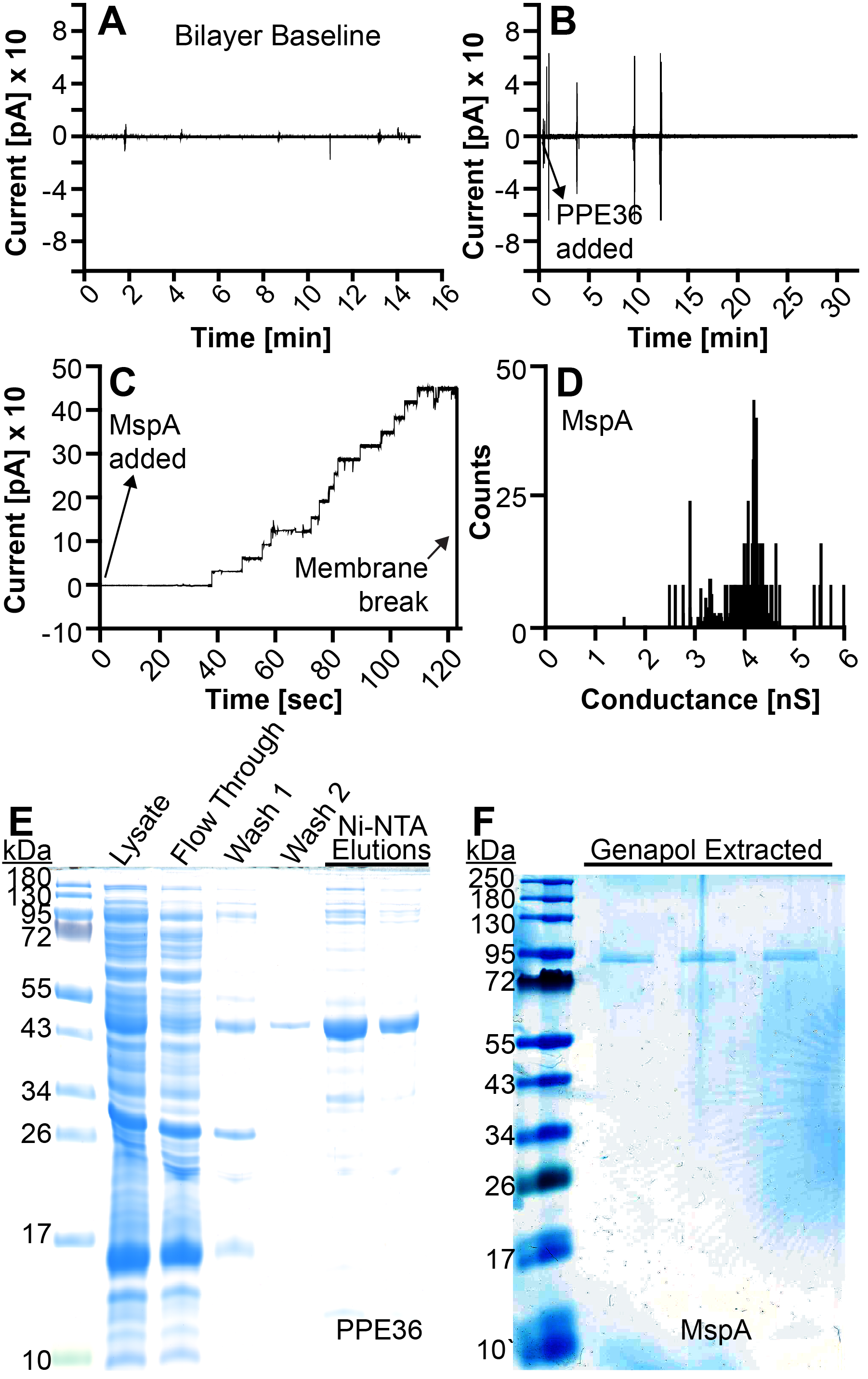

Supplement: FIG S5 [file msphere.00573-22-s0005.tif]
