# Supplementary material for: Role of the Mycobacterium tuberculosis ESX-4 Secretion System in Heme Iron Utilization and Pore Formation by PPE Proteins
Source: mSphere. 2023 Feb 7;8(2):e00573-22. doi: 10.1128/msphere.00573-22 (PMC10117145; doi:10.1128/msphere.00573-22)
Supplement: TABLE S3 [file msphere.00573-22-s0009.docx]

**STRAINS USED IN THIS WORK**

| **Strain** | **Parent strain and relevant genotype** | **Source** |
| --- | --- | --- |
| *E. coli* DH5α | *recA1; endA1; gyrA96; thi; relA1; hsdR17(rK-;mK+); supE44; φ80*Δ*lacZ*Δ*M15;* Δ*lacZYA-argF; UE169* |  |
| *M. smegmatis* mc^2^155 | wild-type (for MspA extraction) | ATCC# 700084 |
| *M. tuberculosis* H37Rv | wild-type | ATCC# 25618 |
| *M. tuberculosis* OAL103 | Δ*eccC4*::*loxP* | This study |
| *M. tuberculosis* OAL104 | Δ*eccC4*::*loxP*; pOAL102; hyg^R^ (*eccC4* complement strain) | This study |
| *M. tuberculosis* OAL105 | Δ*rv0125*::*hyg*; hyg^R^ | This study |
| *M. tuberculosis* OAL106 | Δ*rv1085*::*hyg*; hyg^R^ | This study |
| *M. tuberculosis* OAL107 | Δ*sigM*::*hyg*; hyg^R^ | This study |
| *M. tuberculosis* OAL111 | wt ; pOAL311; hyg^R^ (wt expressing *hs1-M7A* heme biosensor) | This study |
| *M. tuberculosis* OAL112 | wt ; pOAL313; hyg^R^ (wt expressing *ppe36*_HA_) | This study |
| *M. tuberculosis* OAL113 | wt ; pOAL314; hyg^R^ (wt expressing *ppe62*_HA_) | This study |
| *M. tuberculosis* OAL114 | Δ*eccC4*::*loxP* ; pOAL313; hyg^R^ (wt expressing *ppe36*_HA_) | This study |
| *M. tuberculosis* OAL115 | Δ*eccC4*::*loxP*; pOAL314; hyg^R^ (wt expressing *ppe62*_HA_) | This study |
| *M. tuberculosis* OAL118 | wt ; pOAL317; hyg^R^ (wt expressing *ppe64*_HA_) | This study |
| *M. tuberculosis* OAL121 | Δ*eccC4*::*loxP*; pOAL317; hyg^R^ (Δ*eccC4* expressing *ppe64*_HA_) | This study |
| *M. tuberculosis* OAL125 | Δ*rv0125*::*hyg*; pOAL319; kan^R^ (Δ*rv0125*::*hyg* expressing *rv0125*) | This study |
| *M. tuberculosis* OAL126 | Δ*rv1085c*::*hyg*; pOAL320; kan^R^ (Δ*rv1085c*::*hyg* expressing *rv1085c*) | This study |
| *M. tuberculosis* OAL127 | Δ*sigM*::*hyg*; pOAL321; kan^R^ (Δ*sigM*::*hyg* expressing *sigM*) | This study |

The annotation hyg^R^ and kan^R^ indicate that the strain is resistant to the antibiotics hygromycin and kanamycin, respectively.

**PLASMIDS USED IN THIS WORK**

| **Parent Vectors** | **Description** | **Marker** |
| --- | --- | --- |
| pML2424 | parent vector for construction of KOs in Mycobacteria by homologues recombination; HygR | Hyg |
| pET21a+ | Plasmid used for 6His-tagged protein purification | Amp |
| pML1970 | Plasmid used for 6His-MBP-tagged protein purification | Amp |
| pMN016 | cloning vector for expression of mycobacterial genes under strong psmyc promoter using PacI-HindIII restriction sites | Hyg |
| pML2714 | Cre recombinase vector for excision of gfp-hyg cassette utilizing loxP sites | Kan |
| pML1335 | mycobacterial attP site integrative cloning vector for chromosomal integration and expressing genes from psmyc promoter | Hyg |
| pML2300 | mycobacterial attP site integrative cloning vector for chromosomal integration and expressing genes from psmyc promoter | Kan |

| **Gene Deletion Vectors** | **Description** | **Marker** |
| --- | --- | --- |
| pOAL101 | 1000bp upstream (*Spe*I-*Swa*I) & downstream (*Pac*I-*Nsi*I) of *eccC4* cloned into pML2424 (This is the knockout plasmid for deletion of *eccC4*) | Hyg |
| pOAL105 | 1000bp upstream (*Spe*I-*Swa*I) & downstream (*Pac*I-*Nsi*I) of *rv0125* cloned into pML2424 (This is the knockout plasmid for deletion of *rv0125*) | Hyg |
| pOAL106 | 1000bp upstream (*Spe*I-*Swa*I) & downstream (*Pac*I-*Nsi*I) of *rv1085* cloned into pML2424 (This is the knockout plasmid for deletion of *rv1085*) | Hyg |
| pOAL107 | 1000bp upstream (*Spe*I-*Swa*I) & downstream (*Pac*I-*Nsi*I) of *sigM* cloned into pML2424 (This is the knockout plasmid for deletion of *sigM*) | Hyg |

| **Expression Vectors** | **Description** | **Marker** |
| --- | --- | --- |
| pOAL102 | *eccC4* cloned into pML1335 (integrative vector for chromosomal expression vector) | Hyg |
| pOAL311 | *hs1-M7A* cloned into pMN016 (episomal expression vector, smyc promoter) | Hyg |
| pOAL313 | *pe22-ppe36*_HA_ cloned into pML1335 (C-term PPE36_HA_ integrative chromosomal exp. vector, smyc promoter) | Hyg |
| pOAL314 | *ppe62*_HA_ cloned into pM1335 (C-term PPE62_HA_ integrative chromosomal exp. vector, smyc promoter) | Hyg |
| pOAL317 | *ppe64*_HA_ cloned into pML1335 (C-term PPE64_HA_ integrative chromosomal exp. vector, smyc promoter) | Hyg |
| pOAL319 | *rv0125* cloned into pML2300 (integrative chromosomal expression vector, smyc promoter) | Kan |
| pOAL320 | *rv1085c* cloned into pML2300 (integrative chromosomal expression vector, smyc promoter) | Kan |
| pOAL321 | *sigM* cloned into pML2300 (integrative chromosomal expression vector, smyc promoter) | Kan |

| **Protein purification vectors** | **Description** | **Marker** |
| --- | --- | --- |
| pOAL301 | *ppe64* cloned into *Nde*I-*Xho*I digested pET21a+ | Amp |
| pOAL306 | *ppe51* cloned into *Xba*I-*Xho*I digested pET21a+ | Amp |
| pOAL312 | *ppe38* cloned into *Nde*I-*Hind*III digested pET21a+ | Amp |
| pOAL201 | *mhuD* cloned into *Nde*I-*Hind*III digested pET21a+ | Amp |
| pOAL202 | *ideR* cloned into *Nde*I-*Hind*III digested pET21a+ | Amp |
| pOAL203 | *pe22-ppe36* cloned in to *NdeI-XhoI* digest pET21a+ | Amp |

The annotations hyg, kan and amp indicate resistance to the antibiotics hygromycin, kanamycin and ampicillin, respectively.

**PRIMERS USED IN THIS WORK**

| ***Real Time Primers*** | |  | ***Real Time Primers*** | |
| --- | --- | --- | --- | --- |
| rv3446-RT/F | atctcgaccacgctgcta |  | rv3175-RT/R | ccatttgagttggtcccagt |
| rv3446-RT/R | ATGACGCGGGGTATTACC |  | rv0374-RT/F | gcaggtgaacatgacggtaa |
| eccC4-RT/F | caggcacattcgataggaca |  | rv0374-RT/R | tatcacagccccagtga |
| eccC4-RT/R | aattcgtcgacgtgttcctc |  | ppe38-RT/F | aacatcttcgggcagaacac |
| eccD4-RT/F | GGTGTTTGCCATCTGTGGAA |  | ppe38-RT/R | tcagtgaacgctgtcattcg |
| eccD4-RT/R | AGCGACGAAGCCCAAAAACA |  | ppe51-RT/F | cgggacgaactacaacaa |
| mycP4-RT/F | AACCCTCGGCATTCACTCT |  | ppe51-RT/R | ggcaggaccatttttgactc |
| mycP4-RT/R | ATCGGAATCGAACCATGCTG |  | rv1574-RT/F | gaactcctgggttccaaaca |
| eccB4-RT/F | acctggaataccgagcagtg |  | rv1574-RT/R | acgtcgttcataccgttggt |
| eccB4-RT/R | actctacgtgcgagtgga |  | embR-RT/F | acagctgatcaccgcctact |
| rv2254c-RT/F | caacgtgttgagggtttgg |  | embR-RT/R | cagaatccgctcgttgagag |
| rv2254c-RT/R | cacagcccagctgtttttgt |  | mesT-RT/F | cgcatctacctggactaca |
| lprA-RT/F | gcgatatctccaacacac |  | mesT-RT/R | gagtagtcgaaggcaacga |
| lprA-RT/R | tggcctaggtcggagtac |  | rv3054-RT/F | ctgccgttctacaacgaaga |
| rv3174-RT/F | cgcaaagtggcaaaggtcta |  | rv3054-RT/R | gaatgctgccgttgtattcc |
| rv3174-RT/R | cgacatcggttgctaagt |  | 16S-RT/F | TGCTACAATGGCCGGTACAAA |
| rv3175-RT/F | tcttcaacgagttcgacgtg |  | 16S-RT/F | GCGATTACTAGCGACGCCGACTT |

| ***Gene Deletion Primers*** | |  | ***Gene Deletion Primers*** | |  |
| --- | --- | --- | --- | --- | --- |
| eccC4-KO-LF/SpeI | atatACTAGTccggccggtgacgcgtcgat |  | rv0125-RR/NsiI | atatATGCATCGGTGTTGGGATCACCGAAA | |
| eccC4-KO-LR/SwaI | atatATTTAAATGCCCACATCGCGCGGTGATC |  | rv1085-LF/SpeI | atatACTAGTCCGACCCCGACGACGCGGCG | |
| eccC4-KO-RF/PacI | atatTTAATTAAgctgggcgacggtatgcagc |  | rv1085-LR/SwaI | atatATTTAAATCCCGGCACGCCCAGGGCGGT | |
| eccC4-KO-RR/NsiI | atatATGCATGGGTTCAGCGCGGGCGGCAA |  | rv1085-RF/PacI | atatTTAATTAATTCGCCTGTTGCCTCGTCTT | |
| sigM-KO-LF/SpeI | atatACTAGTGGCATCGGCGCCTCCCTGTA |  | rv1085-RR/NsiI | atatATGCATCACCGGGTTTCACCTGGGCA | |
| sigM-KO-LR/SwaI | atatATTTAAATAGCACTTCACCCTGGCTGGC |  | rv0125-V/F | CGGAGACAGCAGTTTCGCTAA | |
| sigM-KO-RF/PacI | atatTTAATTAACGGACACTGGGGCCGATGAG |  | rv0125-V/R | CTGTGTTCTGCCTCGTTCAT | |
| sigM-KO-RR/NsiI | atatATGCATAGACCAGCGGCGCCAGCTGG |  | rv1085-V/F | CTACCGGATTGCTGGACAC | |
| rv0125-LF/SpeI | atatACTAGTCACGGCGGTGCGGGCGGTGC |  | rv1085-V/R | CGGTACAACGGCTCTTTGA | |
| rv0125-LR/SwaI | atatATTTAAATCCCGTAACCTCCTGTGTCGG |  | sigM-V/F | GAAAGAGTCAAGCCGACATC | |
| rv0125-RF/PacI | atatTTAATTAATTTCGTCGCGGATACCACCCG |  | sigM-V/R | TTCAACGCGCGCAGGATTT | |

|  | ***Gene Expression Primers*** | |
| --- | --- | --- |
|  | eccC4-1335/F-PacI | atatTTAATTAAATGAATTCAGGGCCGGCGTGCGCG |
|  | eccC4-1335/R-SwaI | atatATTTAAATTCACGGGGGTGGGCTCCAGG |
| HS-016Clone/F | | atatATTTAAATCCCGGCACGCCCAGGGCGGT |
|  | HS-016Clone/R | atatTTAATTAATTCGCCTGTTGCCTCGTCTT |
|  | ppe36-HA/F-PacI | atatTTAATTAAGGGAGAACAATGCCCAATTTCTGGGCGTT |
|  | ppe36-HA/R-HindIII | atatAAGCTTtcaggcgtagtccggcacgtcgtacgggtaAAACTTAGGATGTTCCT |
|  | ppe62-HA/F-PacI | atatTTAATTAAGGGAGAACAATGAACTATGCGGTATTGCC |
|  | ppe62-HA/R- HindIII | atatAAGCTTtcaggcgtagtccggcacgtcgtacgggtaGTTCCCGAACCCCGACC |
|  | ppe64-HA/F-PacI | atatTTAATTAAatggctcatttttcggtgtt |
|  | ppe64-HA/R- HindIII | atatAAGCTTtcaggcgtagtccggcacgtcgtacgggtaccccaacagctggcgca |
|  | rv0125-2300/F-PacI | atatTTAATTAAATGCATCGGTGTTGGGATCACCGAAA |
|  | rv0125-2300/R-HindIII | atatACTAGTTCAGGCCGGGGGTCCCTCGG |
|  | rv1085-2300/F-PacI | atatTTAATTAAATGAGCGGCCAGGCCGACACCGC |
|  | rv1085-2300/R-HindIII | atatACTAGTTCAGAACACCACGAACCACA |
|  | sigM-2300/F-PacI | atatTTAATTAAatgccgccaccgattggtta |
|  | sigM-2300/R-HindIII | atatACTAGTtcatgcccggtggcaatagc |

| ***Protein Purification Primers*** | |  |
| --- | --- | --- |
| ppe64-21a-F/NdeI | atatCATATGGCTCATTTTTCGGTGTT |  |
| ppe64-21a-R/XhoI | atatCTCGAGCCCCAACAGCTGGCGCAGGT |  |
| ppe51-21a-F/XbaI | atatTCTAGAaataattttgtttaactttaagaaggagatataatggatttcgcactgttacca | |
| ppe51-21a-R/XhoI | atatCTCGAGccctgccgcgggtgggtggg | |
| ppe38-pET-Nde/F | atatCATATGATTTTGGATTTTTCGTGGTT | |
| ppe38-pET-Hind/R | atataagcttctagtggtggtggtggtggtggtggtgtccgatcccgacccgcggca | |
| mhuD-21a-F/NdeI | atatCATATGCACCACCACCACCACCACCCAGTGGTGAAGATCAACGCAATC | |
| mhuD-21a-R/HindIII | atatACTAGTTTATGCAGTCTTGCCGGTCC |  |
| ideR-21a-Nde/F | atatCATATGCACCACCACCACCACCACAACGAGTTGGTTGATACCACCGAG |  |
| ideR-21a-Hind/R | atatACTAGTTCAGACTTTCTCGACCTTGA |  |
| pe22-ppe36-21a-Nde/F | atatCATATGTCTTTGAGTTTCGAGGAGGAG |  |
| pe22-ppe36-21a-Xho/R | atatCTCGAGAAACTTAGGATGTTCCTTGT |  |
